# Diagnostic triage for ultrasound-detected subclinical insulin injection–related lipohypertrophy in older adults with diabetes: development and internal validation

**DOI:** 10.3389/fendo.2026.1997042

**Published:** 2026-10-21

**Authors:** Shuyi Yu, Yunyu Wang, Weihao Wang, Zhiwen Liu, Quanying Wu

**Affiliations:** 1 Department of Endocrinology, Beijing Hospital, Beijing, China; 2 National Center of Gerontology and Institute of Geriatric Medicine, Chinese Academy of Medical Sciences, Beijing, China; 3 Department of Ultrasound Medicine, Beijing Hospital, Beijing, China; 4 Department of Nursing, Beijing Hospital, Beijing, China

**Keywords:** diabetes mellitus, diagnostic triage, insulin therapy, lipohypertrophy, older adults, prediction model, ultrasonography

## Abstract

**Background:**

Subclinical insulin injection–related lipohypertrophy can remain undetected after routine injection-site examination. We developed and internally validated a model to prioritize confirmatory ultrasound in older adults with diabetes after negative visual inspection and palpation.

**Methods:**

This single-center cross-sectional study included 818 adults aged 60 years or older from the endocrinology outpatient clinics and inpatient wards of Beijing Hospital between January 2025 and January 2026; 129 had ultrasound-detected subclinical lipohypertrophy. An 11-predictor ridge logistic model combined sex, age, body mass index, insulin injection duration, daily injection frequency, needle reuse, site rotation, injection area, immediate injection after refrigeration, glycated hemoglobin, and weekly glucose-variability frequency. It was compared with a 9-predictor core model that excluded the two metabolic markers. Internal validation used 2,000 bootstrap resamples and repeated nested 10-fold cross-fitting. Out-of-bag bootstrap validation assessed thresholds targeting 90% and 95% sensitivity.

**Results:**

Subclinical lipohypertrophy was detected in 15.8% (95% CI, 13.4%–18.4%). The core model had an optimism-corrected receiver operating characteristic (ROC) area under the curve of 0.758 (95% interval, 0.720–0.808) and Brier score of 0.117 (0.100–0.130). The extended model had an optimism-corrected ROC area under the curve of 0.774 (95% interval, 0.741–0.823), precision-recall area under the curve of 0.423 (0.353–0.527), and Brier score of 0.113 (0.097–0.126). At the validated thresholds, mean sensitivities were 89.7% and 94.4%, and 337 and 220 ultrasound examinations per 1,000 patients, respectively, were avoided.

**Discussion:**

The model stratified the probability of ultrasound-detected subclinical lipohypertrophy after a negative routine examination. Its high-sensitivity operating points quantify the balance between ultrasound use and missed lesions, providing a basis for evaluating selective ultrasound referral in older adults receiving insulin. The core model warrants evaluation as a lower-burden option. Temporal and multicenter geographic external validation is required before clinical software deployment or widespread protocol adoption.

## 1 Introduction

Insulin injection–related lipohypertrophy (LH) is a common complication of long-term insulin treatment. Injection into affected tissue is associated with irregular insulin absorption, glycemic variability, hypoglycemia, higher glycated hemoglobin, and higher insulin requirements (1). These problems can prompt repeated dose adjustments while the underlying injection-site lesion remains unrecognized. Older adults face additional difficulties with site rotation, needle replacement, and self-examination because of impaired vision, memory, or dexterity and limited caregiving support (2). Irregular rotation and needle reuse are consistently associated with LH (3). Current injection-technique recommendations therefore incorporate injection-site assessment, LH management, and structured education into routine diabetes care (4).

Visual inspection and palpation can miss lesions that are deep, non-raised, or poorly demarcated. In one prospective cohort, ultrasound identified 41.1% more affected patients and 61.2% more lesions than physical examination (5); Luo et al. detected subclinical LH in 19.9% of all 316 enrolled insulin-treated participants (6). A systematic review of 20 studies and 5,067 participants reported ultrasound-detected proportions of 13%–55% and described nodular, diffuse, and hypoechoic phenotypes (7). High-frequency ultrasound and elastography provide additional information on subcutaneous structure and stiffness (8, 9). Small non-randomized follow-up studies have also documented changes in lesion appearance after site avoidance and correction of injection technique (5, 10).

The clinical question is how to prioritize ultrasound after a negative routine examination when imaging capacity is limited. Detection proportions vary with patient characteristics, scanned sites, and imaging definitions, making the clinical entry population central to model development (7, 11). Surveys in China identify persistent needle reuse, insufficient rotation, knowledge gaps, and practical barriers to injection-site care (12, 13). A previous diagnostic model was developed in a cohort with 89.6% ultrasound positivity (14). We focused on older adults with negative visual inspection and palpation and developed a predefined 11-predictor ridge logistic model to estimate current ultrasound positivity. We compared it with a 9-predictor core model and evaluated discrimination, calibration, prediction error, clinical net benefit, high-sensitivity operating points, and prediction stability. The intended users are clinicians and diabetes nurses arranging confirmatory ultrasound.

## 2 Materials and methods

### 2.1 Study design and participants

This single-center cross-sectional diagnostic prediction-model study consecutively recruited adults aged 60 years or older receiving subcutaneous insulin from the endocrinology outpatient clinics and inpatient wards of Beijing Hospital between January 2025 and January 2026. Assessment comprised standardized visual inspection and palpation, predictor collection, and high-frequency ultrasound in participants with negative routine examinations. The outcome was current ultrasound-detected subclinical insulin injection–related LH.

Of 1,000 patients screened, 182 with injection-site abnormalities on visual inspection or palpation were excluded. These patients were recorded as an aggregate count. The remaining 818 examination-negative participants underwent ultrasound and formed the cohort for model development and internal validation (**Figure 1**). Study size was determined by consecutive recruitment during the study period, and internal validation used resampling of the full cohort.

**Figure 1:**
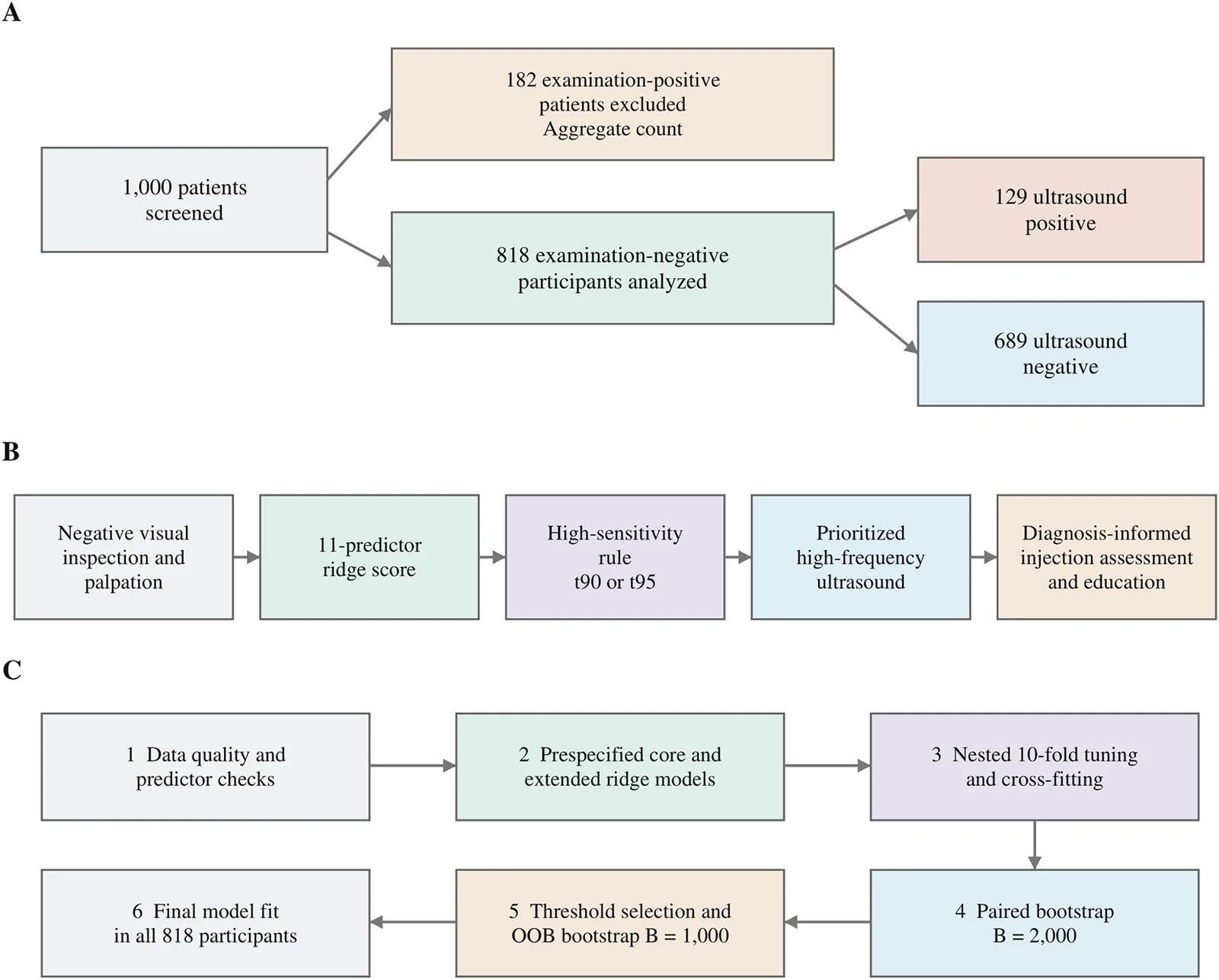
Study cohort, clinical triage pathway, and modeling workflow. **(A)** Participant flow: 1,000 patients were screened; 182 with positive routine examinations were recorded as an aggregate exclusion count; 818 examination-negative participants underwent high-frequency ultrasound, including 129 ultrasound-positive and 689 ultrasound-negative participants. **(B)** Proposed pathway from a negative routine examination through model-guided ultrasound prioritization to diagnosis-informed assessment and education. **(C)** Model development, internal validation, operating-point assessment, and final fitting in all 818 participants.

Eligibility required age 60 years or older; diabetes diagnosed according to the American Diabetes Association Standards of Care in Diabetes—2025 (15) or a documented endocrinologist-established diagnosis under treatment; current subcutaneous insulin treatment; ability to complete the injection-behavior questionnaire and injection-site assessment; and agreement to ultrasound. Exclusions were acute infection, ulceration, severe skin disease, prominent scarring, or another local lesion that may impede ultrasound interpretation; recent surgery, trauma, or edema at the abdomen or another commonly used injection site; severe cognitive or communication impairment precluding reliable injection-behavior information; or missing clinical data or key variables.

The Ethics Committee of Beijing Hospital approved the study (2023BJYYEC-215-01; August 3, 2023). All participants provided written informed consent before study procedures.

### 2.2 Data collection and candidate predictors

Predictors were collected before ultrasound outcome ascertainment. Research nurses reviewed electronic medical records and obtained data through face-to-face interviews, standardized physical measurements, and an injection-behavior questionnaire during the same episode of care. Ultrasound followed visual inspection, palpation, questionnaire completion, and clinical data entry. Predictor collectors were unaware of ultrasound results until records were locked; ultrasound readers had no access to model predictions.

The source database contained 26 variables covering demographics, clinical and laboratory measures, insulin exposure, and injection behavior (**Supplementary Table S1**). Predictor sets were specified in the modeling analysis plan before formal model fitting. The 9-predictor core model comprised sex, age, body mass index, years of insulin injections, daily injection frequency, needle reuse count, regular site rotation, self-reported injection area, and immediate injection after removing insulin from refrigeration. The primary extended model added glycated hemoglobin and weekly glucose-variability frequency to assess the diagnostic information contributed by metabolic markers (1, 3, 4).

Male sex was coded 1 and female sex 0. Daily injection frequency was coded once daily = 1, twice daily = 2, and three times daily = 3. Irregular rotation, a 5 × 8 cm injection area (length × width), and immediate injection after refrigeration were coded 1. Continuous and count predictors were retained on their original scales. Predictor coding and final coefficients are reported in **Table 1**, with the full variable dictionary in **Supplementary Table S1**.

**Table 1:** Primary extended ridge model specification and final coefficients.

Predictor	Model coding	Original-scale coefficient	Standardized coefficient
Intercept	Model intercept	-9.341685	-9.342
Male sex	Male = 1; female = 0	-0.275172	-0.134
Age	Continuous, years	0.036202	0.234
Body mass index	Continuous, kg/m²	0.057255	0.182
Insulin injection duration	Continuous, years	0.048498	0.176
Daily injection frequency	Once daily = 1; twice daily = 2; three times daily = 3	0.298719	0.212
Needle reuse count	Continuous count	0.168354	0.262
Irregular site rotation	Irregular = 1; regular = 0	0.730316	0.344
Small injection area	5 × 8 cm = 1; 10 × 15 cm = 0	0.158505	0.077
Immediate injection after refrigeration	Yes = 1; no = 0	0.721440	0.243
Glycated hemoglobin	Continuous, percentage points	0.235588	0.210
Glucose-variability frequency	Continuous, events/week	0.504010	0.296

Selected lambda = 0.028735. Original-scale coefficients correspond to one raw unit. Coefficients are descriptive parameters of the fitted ridge model.

For a participant with predictor values coded as shown, the linear predictor is the intercept plus the sum of each original-scale coefficient multiplied by its predictor value. The predicted probability is:.

p = 1 / (1 + exp(−η)).

Here, η denotes the linear predictor. For t90 or t95, a probability at or above the development threshold indicates referral for ultrasound.

Glucose-variability frequency was assessed by questionnaire over the preceding 3 months. An event was a glucose value below or above the participant’s individualized glycemic target during otherwise stable glycemic control. Finger-stick measurements or continuous glucose monitoring readings were assessed at the same seven-point glucose time points; symptoms were not required. The recalled number of events was divided by the actual number of weeks in the recall period to obtain the average weekly frequency. All participants had performed glucose monitoring; recall was used to supplement the monitoring history. This self-reported measure is distinct from a continuous glucose monitoring-derived variability metric.

### 2.3 Ultrasound outcome ascertainment

Sites used for long-term insulin injections were scanned. A research nurse used the injection history and questionnaire to mark commonly used, recently repeated, and suspected areas. The sonographer placed the transducer lightly to avoid compressing subcutaneous tissue and performed multipoint longitudinal and transverse scans, with additional assessment of the lesion center and surrounding tissue when required.

Following Wang et al. (5), four features were recorded: a relatively well-defined subcutaneous nodule with a hypoechoic or hyperechoic halo; distortion of surrounding connective-tissue architecture; absence of internal vascular signals and a capsule; and heterogeneous echotexture relative to surrounding tissue. A reproducible lesion meeting at least three features was ultrasound positive. A lesion satisfying these criteria but not identified as definite LH on visual inspection or palpation was classified as subclinical LH. Two experienced sonographers interpreted examinations independently and were mutually blinded; disagreements were adjudicated by a third senior sonographer.

### 2.4 Model development and internal validation

Data checks covered dimensions, unique identifiers, variable types, allowed levels, outcome counts, missingness, and ranges. The source worksheet contained no missing values, and all observations were retained after range checks. Model matrices contained the prespecified predictors; study identifiers, an outcome-duplicating descriptive group, and the remaining descriptive variables were excluded.

Core and extended models used ridge logistic regression (glmnet; family = binomial; alpha = 0) with an intercept and internal standardization. Within each training or resampled dataset, stratified 10-fold cross-validation selected lambda.min by minimum binomial deviance. Models used unweighted observations at the observed class proportions. After internal validation, lambda was reselected and each final model was fitted in all 818 participants.

Optimism in the complete modeling procedure was estimated with 2,000 paired ordinary bootstrap resamples. Design-matrix construction, inner folds, penalty selection, model fitting, prediction, calibration, and scoring were repeated within each resample; assessment observations were used only for prediction and scoring. Core and extended models shared indices for paired comparisons. Performance measures comprised the receiver operating characteristic (ROC) area under the curve (AUC), precision-recall AUC, average precision, Brier score, scaled Brier score, log loss, calibration intercept and slope, and absolute calibration errors. Most uncertainty intervals used location-shifted percentile bootstrap estimates. Calibration-intercept and slope distributions used a separate two-stage bootstrap with 200 outer and 500 inner resamples (16).

Patient-level out-of-fold probabilities were generated through repeated nested cross-fitting with stratified 10-fold outer splits repeated 20 times. Inner stratified 10-fold cross-validation selected lambda within each outer training set; predictions were generated only for held-out observations and averaged across repeats. These probabilities supported ROC and precision-recall curves, risk distributions, calibration by risk decile, decision curves, threshold development, and stability analyses (**Figure 2**). Full resampling details are provided in **Supplementary Methods S2**.

**Figure 2:**
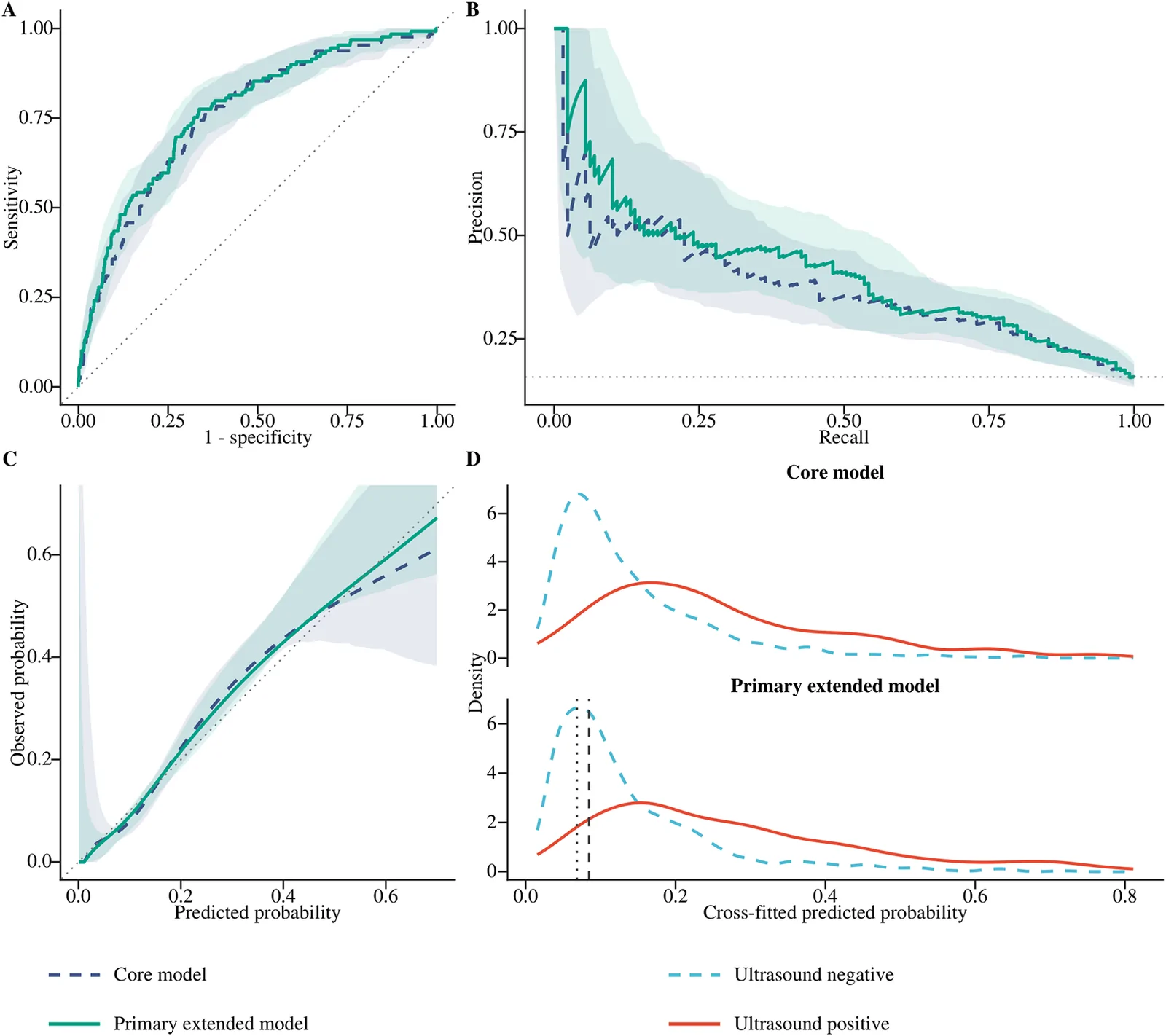
Discrimination, calibration, and predicted probability distributions. **(A)** Receiver operating characteristic (ROC) curves. **(B)** Precision-recall curves. **(C)** Optimism-corrected calibration curves with bootstrap uncertainty. **(D)** Distributions of repeated nested cross-fitted probabilities by ultrasound outcome, with the t90 and t95 development thresholds indicated. ROC and precision-recall curves use repeated nested cross-fitted probabilities.

### 2.5 Clinical utility and operating-point assessment

Decision-curve analysis treated referral for high-frequency ultrasound as the positive decision and compared the extended model, core model, refer-all, and refer-none strategies across threshold probabilities of 0.02–0.30 in increments of 0.005. Five hundred patient-level bootstrap resamples quantified uncertainty (17, 18). Net benefit summarized the balance between identifying ultrasound-positive patients and referring ultrasound-negative patients within this range.

For target sensitivities of 0.90 and 0.95, the highest unique thresholds attaining each target in repeated cross-fitted predictions were selected as t90 and t95. The complete selection procedure was validated with 1,000 bootstrap resamples. Training, tuning, cross-fitting, and threshold selection were repeated in-bag; a model fitted in-bag predicted unique out-of-bag (OOB) participants, whose outcomes were used only to evaluate the fixed threshold. We reported sensitivity, specificity, predictive values, referral proportions, and consequences per 1,000 patients.

### 2.6 Statistical analysis

Continuous variables with absolute skewness of 1 or less were summarized as mean (SD), otherwise as median [first quartile, third quartile]; categorical variables were summarized as n (%). Standardized mean differences and prespecified correlation audits described the cohort. Prespecified sensitivity analyses used lambda.1se, modeled injection frequency as a factor, fitted unpenalized logistic regression with the same 11 predictors, used three-degree-of-freedom natural splines for insulin duration, glycated hemoglobin, and glucose variability, and applied robust scaling. All data-dependent transformations remained within training folds.

Analyses, tables, and figures were generated in R 4.6.0 using targets 1.12.0, renv 1.2.3, and glmnet 5.0. The global seed was 20260720. Reporting followed Transparent Reporting of a multivariable prediction model for Individual Prognosis Or Diagnosis + Artificial Intelligence (TRIPOD+AI) (19). The modeling analysis plan was local and was not publicly deposited.

## 3 Results

### 3.1 Participant characteristics and subclinical LH detection

Among 1,000 screened patients, 182 were excluded because visual inspection or palpation identified an injection-site abnormality. All 818 remaining participants underwent ultrasound: 129 were positive and 689 negative, yielding a subclinical LH detection proportion of 15.8% (95% CI, 13.4%–18.4%) (**Figure 1**). All identifiers were unique and all 26 source variables were complete.

Mean age was 73.17 (SD, 6.46) years, 497 (60.8%) participants were women, and mean body mass index was 24.75 (SD, 3.18) kg/m². Ultrasound-positive participants were older and had descriptively higher body mass index, glycated hemoglobin, insulin injection duration, needle reuse, and glucose-variability frequency, with less regular site rotation and more frequent injection in a small area or immediately after refrigeration (**Table 2**; **Supplementary Figure S1**).

**Table 2:** Cohort characteristics by current ultrasound outcome.

Characteristic	All participants (n = 818)	Ultrasound negative (n = 689)	Ultrasound positive (n = 129)
Sex, n (%): Female	497 (60.8%)	411 (59.7%)	86 (66.7%)
Sex, n (%): Male	321 (39.2%)	278 (40.3%)	43 (33.3%)
Age, years	73.17 (6.46)	72.77 (6.34)	75.33 (6.70)
Body mass index, kg/m²	24.75 (3.18)	24.59 (3.14)	25.61 (3.22)
Insulin injection duration, years	6.61 (3.62)	6.39 (3.56)	7.82 (3.69)
Daily injection frequency, n (%): Once daily	247 (30.2%)	222 (32.2%)	25 (19.4%)
Daily injection frequency, n (%): Twice daily	399 (48.8%)	339 (49.2%)	60 (46.5%)
Daily injection frequency, n (%): Three times daily	172 (21.0%)	128 (18.6%)	44 (34.1%)
Needle reuse count	0.00 [0.00, 2.00]	0.00 [0.00, 2.00]	2.00 [0.00, 3.00]
Regular site rotation, n (%): Yes	546 (66.7%)	490 (71.1%)	56 (43.4%)
Regular site rotation, n (%): No	272 (33.3%)	199 (28.9%)	73 (56.6%)
Self-reported injection area, n (%): 10 × 15 cm	503 (61.5%)	436 (63.3%)	67 (51.9%)
Self-reported injection area, n (%): 5 × 8 cm	315 (38.5%)	253 (36.7%)	62 (48.1%)
Immediate injection after refrigeration, n (%): No	711 (86.9%)	615 (89.3%)	96 (74.4%)
Immediate injection after refrigeration, n (%): Yes	107 (13.1%)	74 (10.7%)	33 (25.6%)
Glycated hemoglobin, %	7.45 (0.89)	7.38 (0.87)	7.81 (0.95)
Glucose-variability frequency, events/week	0.00 [0.00, 0.60]	0.00 [0.00, 0.60]	0.40 [0.00, 1.20]

Values are mean (SD), median [first quartile, third quartile], or n (%), according to the predefined summary rule.

### 3.2 Model development and performance

The selected full-data lambda was 0.023857 for the core model and 0.028735 for the primary extended model. In the extended model, the largest absolute standardized coefficients were for irregular site rotation (0.344), glucose-variability frequency (0.296), needle reuse count (0.262), immediate injection after refrigeration (0.243), and age (0.234). **Table 1**; **Supplementary Figure S2** present the model coefficients.

After bootstrap optimism correction, ROC AUCs were 0.758 (95% interval, 0.720–0.808) for the core model and 0.774 (0.741–0.823) for the extended model. Corresponding precision-recall AUCs were 0.386 (0.316–0.489) and 0.423 (0.353–0.527), and Brier scores were 0.117 (0.100–0.130) and 0.113 (0.097–0.126). For the extended model, the mean predicted probability equaled the observed proportion (0.158), the calibration intercept was 0.004 (−0.005 to 0.014), and mean absolute calibration error was 0.013 (0.013–0.037) (**Table 3A**; **Figure 2**).

**Table 3:** Internally validated performance and high-sensitivity operating points.

Panel A. Optimism-corrected model performance		
Metric	Core model	Primary extended model
ROC area under the curve	0.758 (0.720 to 0.808)	0.774 (0.741 to 0.823)
Precision-recall area under the curve	0.386 (0.316 to 0.489)	0.423 (0.353 to 0.527)
Brier score	0.117 (0.100 to 0.130)	0.113 (0.097 to 0.126)
Calibration intercept	0.004 (-0.005 to 0.014)	0.004 (-0.005 to 0.014)
Calibration slope	1.072*	1.074*
Mean absolute calibration error (Eavg)	0.018 (0.013 to 0.043)	0.013 (0.013 to 0.037)

Panel B. Out-of-bag validation of the operating-point selection procedure		
Operating point	t90	t95
Development threshold	0.084466	0.068444
Sensitivity	89.7% (77.4% to 98.2%)	94.4% (83.7% to 100.0%)
Specificity	38.1% (22.2% to 53.3%)	25.1% (8.5% to 42.1%)
Positive predictive value	21.5% (16.5% to 26.5%)	19.2% (14.9% to 23.8%)
Negative predictive value	95.4% (91.6% to 99.0%)	96.5% (92.2% to 100.0%)
Referred per 1,000	663 (517 to 810)	780 (619 to 924)
Avoided per 1,000	337 (190 to 483)	220 (76 to 381)
Missed cases per 1,000	16 (3 to 37)	9 (0 to 26)

*Calibration-slope point estimates are shown separately from the two-stage bootstrap distributions: core median 1.213 (95% percentile interval, 1.107–1.437) and extended median 1.241 (1.134–1.400).

Development thresholds are the full-cohort thresholds. Accuracy and clinical-consequence estimates are means with 95% intervals from out-of-bag internal validation.

Paired extended-minus-core differences were 0.015 (95% interval, −0.010 to 0.031) for ROC AUC, 0.037 (−0.011 to 0.070) for precision-recall AUC, and −0.003 (−0.006 to 0.001) for Brier score. Intervals included zero, leaving the magnitude of incremental information from glycated hemoglobin and glucose variability uncertain. The primary model comprised 11 predictors (**Supplementary Table S3**).

### 3.3 Clinical utility and operating points

Extended-model decision-curve point estimates were above refer-all and refer-none strategies from threshold probabilities of 0.035 to 0.30. Net benefit at threshold probabilities of 0.05, 0.10, and 0.20 was 0.116, 0.087, and 0.046, respectively (**Supplementary Figure S3**).

The t90 development threshold was 0.084466. OOB validation yielded mean sensitivity of 89.7% (95% interval, 77.4%–98.2%), specificity of 38.1% (22.2%–53.3%), positive predictive value of 21.5% (16.5%–26.5%), and negative predictive value of 95.4% (91.6%–99.0%). Per 1,000 patients, 663 would be referred, 337 examinations avoided, and 16 cases missed.

The t95 development threshold was 0.068444. Mean OOB sensitivity was 94.4% (83.7%–100.0%), specificity 25.1% (8.5%–42.1%), positive predictive value 19.2% (14.9%–23.8%), and negative predictive value 96.5% (92.2%–100.0%). Per 1,000 patients, 780 would be referred, 220 examinations avoided, and 9 cases missed (**Table 3B**; **Supplementary Table S4**, **Supplementary Figure S4**). Moving from t90 to t95 added approximately 117 examinations and reduced missed cases by 7.4 per 1,000.

### 3.4 Sensitivity analyses

Across lambda.1se, factor coding of injection frequency, unpenalized logistic regression, natural splines, and robust scaling, cross-fitted ROC AUCs for extended models ranged from 0.764 to 0.771 and Brier scores from 0.114 to 0.117, compared with 0.769 and 0.114 for the primary extended model. All observations were retained (**Supplementary Table S5**).

## 4 Discussion

This study addresses ultrasound triage in older adults whose injection sites appear normal on visual inspection and palpation. Ultrasound detected subclinical LH in 15.8% of this population. The extended ridge model distinguished current ultrasound positivity with an optimism-corrected ROC AUC of 0.774 and a Brier score of 0.113. Its two high-sensitivity operating points translated model performance into ultrasound referrals and missed lesions, providing a practical basis for evaluating selective imaging after routine assessment.

The observed detection proportion supports the clinical relevance of lesions missed by routine examination. Wang et al. found that ultrasound identified 41.1% more affected patients and 61.2% more lesions than physical examination (5), and Luo et al. reported subclinical LH in 19.9% of all 316 enrolled insulin-treated participants (6). Our 15.8% estimate is conditional on a negative routine examination, so the differing entry populations and denominators preclude a direct prevalence comparison (6, 7). Patient age, insulin exposure, body habitus, scanned sites, and imaging criteria can also alter the lesion spectrum. Ultrasound phenotypes may have different courses: in a small 6-month non-randomized follow-up, nodular lesions resolved more often than diffuse or hypoechoic lesions after site avoidance and technique correction (10). These differences make consistent outcome ascertainment important when comparing cohorts.

The strongest standardized predictive signals linked injection behavior and glycemic status. Irregular rotation, glucose-variability frequency, needle reuse, immediate injection after refrigeration, and age had the largest absolute standardized coefficients. Associations of LH with irregular rotation, needle reuse, longer insulin exposure, and more frequent injections are also supported by meta-analysis (3). These variables connect the triage assessment to injection-technique review, a central component of current recommendations (4). Immediate injection after refrigeration may reflect broader knowledge or adherence concerning insulin storage and injection procedures, while age may capture differences in function, treatment exposure, and caregiving. The model combines these signals to estimate diagnostic probability.

Adding glycated hemoglobin and glucose-variability frequency produced paired differences of 0.015 in ROC AUC, 0.037 in precision-recall AUC, and −0.003 in Brier score. The corresponding 95% intervals included zero, leaving the size of the incremental contribution uncertain. The clinical rationale for these markers is supported by the co-occurrence of LH with higher glycated hemoglobin, glycemic variability, hypoglycemia, and insulin requirements (1). Their overlap with treatment exposure and injection behavior may explain why they add relatively little information to the core predictor set. The core model warrants evaluation for rapid primary care or nursing triage when metabolic information is unavailable; the extended model may be evaluated when these data are available, including in specialist care. The present estimates do not establish equivalence or clinical preference. This comparison identifies a question for external validation: whether the additional metabolic information improves triage sufficiently to justify its collection in a given care setting.

Clinical entry criteria distinguish this model from earlier diagnostic work. Li et al. studied 395 broadly selected insulin-treated patients, including 354 ultrasound-positive patients, and reported an AUC of 0.756 (14). Our model applies after a negative routine examination, where ultrasound positivity was 15.8%. This difference in clinical spectrum changes the interpretation of predictive values and referral thresholds. The precision-recall AUC of 0.423, calibration measures, and Brier score complement ROC discrimination in describing performance in this setting. External evaluation should assess both probability calibration and the consequences of applying the fixed operating points under local ultrasound practice.

The two operating points offer different balances between imaging use and missed lesions. At t90, the mean OOB sensitivity was 89.7%, with 663 referrals, 337 examinations avoided, and 16 missed lesions per 1,000 patients. At t95, mean sensitivity was 94.4%, with 780 referrals, 220 examinations avoided, and 9 missed lesions per 1,000. Moving from t90 to t95 required about 117 additional examinations for 7.4 fewer missed cases per 1,000. Selection between these operating points depends on ultrasound capacity, the consequences of missed lesions, repeat-assessment feasibility, and patient preferences. Decision-curve point estimates favored selective ultrasound over refer-all and refer-none strategies across threshold probabilities of 0.035–0.30. The relevance of this net benefit depends on local clinical trade-offs (17, 18). When ultrasound confirms LH, assessment and education can address injection sites, rotation, needle use, storage, and injection steps, with caregiver involvement where appropriate (4, 20–23).

Performance was consistent across the prespecified sensitivity analyses, with cross-fitted ROC AUCs of 0.764–0.771 and Brier scores of 0.114–0.117. The median within-participant standard deviation of repeated out-of-fold probabilities was 0.008. Referral classifications nevertheless varied near the operating points: 17.6% of participants had unstable classifications around t90 and 12.0% around t95. In future evaluations, a borderline probability should prompt verification of predictor information, injection-technique review and re-education, and a clinician-led decision on ultrasound or reassessment. This cross-sectional study does not establish a mandatory 3-month reassessment interval.

The single-center, cross-sectional design limits inference to current ultrasound-detected lesions in older insulin-treated adults with negative routine examinations. Self-reported needle reuse, site rotation, injection area, and immediate injection after refrigeration may be affected by recall and social desirability bias. Under-reporting or misclassification may alter predictor weights and calibration, with an uncertain direction of bias, especially if reporting differs across patient groups or care settings. Glucose-variability frequency also depends on recalled monitoring history and individualized glycemic targets. Ultrasound equipment, scanning protocols, and readers may influence model calibration across centers. The 129 positive outcomes also limited precision for small performance differences and threshold estimates. Performance across sociodemographic groups, frailty levels, cognitive function, and caregiving circumstances was not assessed. Temporal and multicenter validation using standardized ultrasound protocols should evaluate calibration, fixed operating points, and subgroup performance. Prospective studies should then compare routine assessment with model-assisted referral to establish its effects on missed lesions, imaging use, and patient outcomes.

## 5 Conclusion

The ridge model stratified the probability of ultrasound-detected subclinical LH in older adults after negative visual inspection and palpation. Its internally validated high-sensitivity operating points quantify the ultrasound use and missed lesions associated with selective referral, defining a clinical pathway for external and prospective evaluation. Temporal and multicenter geographic external validation is required before clinical software deployment or widespread protocol adoption.

## Data Availability

The raw data supporting the conclusions of this article will be made available by the authors, without undue reservation.
