# Supplementary material for: Diagnostic triage for ultrasound-detected subclinical insulin injection–related lipohypertrophy in older adults with diabetes: development and internal validation
Source: Front Endocrinol (Lausanne). 2026 Oct 21;17:1997042. doi: 10.3389/fendo.2026.1997042 (PMC13730916; doi:10.3389/fendo.2026.1997042)
Supplement: Supplementary file 1 [file DataSheet1.pdf]

Supplementary material

Diagnostic Triage for Ultrasound-Detected Subclinical Insulin Injection–Related Lipohypertrophy in Older Adults With Diabetes: Development and Internal Validation

Shuyi Yu, Yunyu Wang, Weihao Wang, Zhiwen Liu, and Quanying Wu

Correspondence: Quanying Wu | wuquanying7659@126.com

Supplementary Methods S1. Outcome ascertainment and ultrasound protocol

Participants whose long-term injection sites were negative on visual inspection and palpation underwent high-frequency ultrasound after predictor collection. A research nurse used the injection history and questionnaire to mark commonly used, recently repeated, and suspected injection areas. The sonographer placed the transducer lightly on the skin to limit compression of subcutaneous tissue and performed multipoint longitudinal and transverse scans. When needed, the lesion center and surrounding tissue were scanned in additional planes.

Four imaging features were assessed according to Wang et al: a relatively well-defined nodule in the subcutaneous layer with a hypoechoic or hyperechoic halo; distortion of surrounding connective-tissue architecture; absence of internal vascular signals and a capsule; and heterogeneous echotexture compared with surrounding tissue. A reproducible lesion meeting at least 3 of 4 features was classified as ultrasound positive. A qualifying lesion not identified as definite lipohypertrophy by visual inspection or palpation was classified as subclinical lipohypertrophy.

Two experienced sonographers interpreted each examination independently and were mutually blinded. Concordant readings formed the report; disagreements were adjudicated by a third senior sonographer. Predictor collectors were unaware of the ultrasound result until records were locked, and ultrasound readers had no access to model predictions.

Ultrasound reference: Wang W, Huang R, Chen Y, Tu M. Values of ultrasound for diagnosis and management of insulin-induced lipohypertrophy: a prospective cohort study in China. Medicine (Baltimore). (2021) 100(29):e26743. doi: 10.1097/MD.0000000000026743

Supplementary Table S1. Complete variable definitions, coding, source, and timing

Variable	Internal field	Unit	Operational definition and coding	Analysis role	Source and timing
Study identifier	id		Integer 1-818; identifier only; excluded from all model matrices	Excluded identifier	Same-visit interview and/or medical record before ultrasound
Descriptive group	group_desc		Ultrasound negative/subclinical lipohypertrophy; duplicates the outcome; excluded from modeling	Excluded outcome duplicate	Same-visit interview and/or medical record before ultrasound
Ultrasound-detected subclinical lipohypertrophy	outcome		0=no; 1=yes; current diagnostic state established by high-frequency ultrasound	Diagnostic outcome	High-frequency ultrasound after predictor collection; two independent readers with senior adjudication
Sex	sex		Female=0; male=1	Core predictor	Same-visit interview and/or medical record before ultrasound
Age	age	years	Continuous years; retained on the original scale	Core predictor	Same-visit interview and/or medical record before ultrasound
Body mass index	bmi	kg/m²	Continuous kg/m² ; retained on the original scale	Core predictor	Same-visit interview and/or medical record before ultrasound
Waist circumference	waist	cm	Continuous cm; retained for description only	Descriptive only	Same-visit interview and/or medical record before ultrasound
Diabetes duration	diabetes_duration	years	Continuous years; retained for description only	Descriptive only	Same-visit interview and/or medical record before ultrasound
Glycated hemoglobin	hba1c	%	Continuous percentage points; retained on the original scale	Extended predictor	Same-episode clinical/laboratory record before ultrasound
Triglycerides	triglycerides	mmol/L	Continuous mmol/L; retained for description only	Descriptive only	Same-episode clinical/laboratory record before ultrasound
Estimated glomerular filtration rate	egfr	mL/min/1.73 m2	Continuous mL/min/1.73 m^2; retained for description only	Descriptive only	Same-episode clinical/laboratory record before ultrasound
C-reactive protein	crp	mg/L	Continuous mg/L; retained for description only	Descriptive only	Same-episode clinical/laboratory record before ultrasound
Urine albumin	urine_albumin	mg/L	Continuous mg/L; retained for description only	Descriptive only	Same-episode clinical/laboratory record before ultrasound
Insulin injection duration	insulin_duration	years	Continuous years; retained on the original scale	Core predictor	Same-visit interview and/or medical record before ultrasound
Total daily insulin dose	insulin_daily_dose	U/day	Continuous units/day; retained for description only	Descriptive only	Same-visit interview and/or medical record before ultrasound
Daily injection frequency	injection_frequency		Once daily=1; twice daily=2; three times daily=3 in the primary models	Core predictor	Standardized injection-behavior questionnaire before ultrasound

Variable	Internal field	Unit	Operational definition and coding	Analysis role	Source and timing
Needle reuse count	needle_reuse_count	uses	Count; retained on the original scale	Core predictor	Standardized injection-behavior questionnaire before ultrasound
Regular site rotation	regular_rotation		Regular=0; irregular=1 in the model	Core predictor	Standardized injection-behavior questionnaire before ultrasound
Self-reported injection area	injection_area	cm × cm (length × width)	10 × 15 cm=0; 5 × 8 cm=1 in the model	Core predictor	Standardized injection-behavior questionnaire before ultrasound
Post-injection leakage	post_injection_leakage		No/yes; retained for description only	Descriptive only	Standardized injection-behavior questionnaire before ultrasound
Immediate injection after refrigeration	immediate_after_refrigeration		No=0; yes=1	Core predictor	Standardized injection-behavior questionnaire before ultrasound
Most recently used needle length	needle_length	mm	Continuous mm; retained for description only	Descriptive only	Standardized injection-behavior questionnaire before ultrasound
Glucose-variability frequency	glucose_variability_freq	events/week	Self-reported events outside individualized glycemic targets over the preceding 3 months, divided by the actual number of weeks; symptoms not required; retained as a continuous average frequency	Extended predictor	Questionnaire before ultrasound; recalled finger-stick or CGM readings at seven-point glucose time points; all participants had monitored glucose
Symptomatic glucose <3.9 mmol/L frequency	symptomatic_hypoglycemia_freq	events/week	Count of self-reported events/week; retained for description only	Descriptive only	Standardized injection-behavior questionnaire before ultrasound
Hypertension	hypertension		No/yes; retained for description only	Descriptive only	Same-visit interview and/or medical record before ultrasound
Dyslipidemia	dyslipidemia		No/yes; retained for description only	Descriptive only	Same-visit interview and/or medical record before ultrasound

Supplementary Methods S2. Complete modeling, internal validation, and threshold procedures

The core and extended models used ridge logistic regression with family=binomial, alpha=0, an intercept, and internal standardization. The core model contained 9 predefined predictors; the primary extended model added glycated hemoglobin and weekly glucose-variability frequency. Predictor sets were fixed before formal model fitting. Continuous and count predictors retained their original scales, and models used unweighted observations at the observed class proportions. Model matrices excluded study identifiers, the outcome-duplicating descriptive group, and variables outside the predefined predictor sets.

Within every training or resampled dataset, deterministic stratified 10-fold cross-validation selected lambda.min by minimum binomial deviance. The complete development procedure was evaluated in 2,000 paired ordinary bootstrap resamples. Design-matrix construction, any preprocessing, inner folds, tuning, fitting, prediction, calibration, and scoring were repeated within each resample. Assessment observations were used only for prediction and scoring. Core and extended models shared indices for paired comparisons.

Optimism-corrected point estimates used apparent performance minus mean optimism. Most 95% intervals used a location-shifted percentile bootstrap. Calibration-intercept and calibration-slope uncertainty was estimated separately using 200 outer and 500 inner bootstrap resamples; corrected point estimates and two-stage distribution medians/percentile intervals are therefore shown separately. The extended-model Emax point estimate was 0.018. Its location-shifted percentile interval was 0.033–0.223 and did not contain the corrected point estimate because the bootstrap distribution was highly skewed. Emax is therefore reported as a point estimate only for the extended model.

Repeated nested cross-fitting generated patient-level out-of-fold probabilities using stratified 10-fold outer splits repeated 20 times. Each outer training set contained an independent stratified 10-fold search for lambda.min; the fitted model predicted only the corresponding held-out fold. The 20 out-of-fold predictions per participant were averaged. These probabilities supported ROC and precision-recall curves, risk distribution, calibration by decile, decision-curve analysis, threshold development, and prediction-stability assessment.

For t90 and t95, the highest unique threshold attaining the target sensitivity in repeated cross-fitted predictions was selected. One thousand patient-level bootstrap resamples internally validated the complete selection procedure. Each in-bag sample repeated training, tuning, cross-fitting, and threshold selection. A model fitted to the full in-bag sample predicted unique out-of-bag participants, whose outcomes were used only to evaluate the fixed threshold. Decision-curve analysis compared the extended model, core model, refer-all, and refer-none strategies across threshold probabilities 0.02–0.30 in increments of 0.005, with 500 bootstrap resamples.

Predefined sensitivity analyses used lambda.1se; treated injection frequency as a 3-level factor; fitted conventional unpenalized logistic regression with the same 11 predictors; applied natural splines with 3 degrees of freedom to insulin injection duration, glycated hemoglobin, and glucose variability; and used robust scaling for core and extended models. Every data-dependent transformation was estimated only in the corresponding training data.

All analyses were conducted in R 4.6.0. Key versions were targets 1.12.0, testthat 3.3.2, glmnet 5.0, ggplot2 4.0.3, dplyr 1.2.1, tidyr 1.3.2, readxl 1.4.5, checkmate 2.3.4, future 1.70.0, future.apply 1.20.2, renv 1.2.3, ragg 1.5.2, svglite 2.2.2, jsonlite 2.0.0, and knitr 1.51. The global seed was 20260720. The dataset contained 818 unique participant identifiers and complete source values.

Supplementary Table S2. Complete optimism-corrected internal-validation results

Metric	Core model	Primary extended model
ROC area under the curve	0.758 (0.720 to 0.808)	0.774 (0.741 to 0.823)
Precision-recall area under the curve	0.386 (0.316 to 0.489)	0.423 (0.353 to 0.527)
Average precision	0.386 (0.321 to 0.491)	0.424 (0.358 to 0.529)
Brier score	0.117 (0.100 to 0.130)	0.113 (0.097 to 0.126)
Scaled Brier score	0.123 (0.074 to 0.196)	0.149 (0.097 to 0.225)
Log loss	0.381 (0.334 to 0.417)	0.370 (0.323 to 0.406)
Calibration intercept	0.004 (-0.005 to 0.014)	0.004 (-0.005 to 0.014)
Calibration slope	1.072*	1.074*
Mean absolute calibration error (Eavg)	0.018 (0.013 to 0.043)	0.013 (0.013 to 0.037)
Median absolute calibration error (E50)	0.016 (0.006 to 0.040)	0.011 (0.005 to 0.035)
90th percentile absolute calibration error (E90)	0.039 (0.022 to 0.090)	0.030 (0.018 to 0.084)
Maximum absolute calibration error (Emax)	0.086 (0.044 to 0.255)	0.018 [point estimate only]
Observed prevalence	0.158	0.158
Mean predicted probability	0.158 (0.133 to 0.184)	0.158 (0.133 to 0.184)

Values are optimism-corrected estimates with 95% intervals unless stated otherwise. *Calibration-slope point estimates are reported separately from two-stage bootstrap distributions: core median 1.213 (95% percentile interval, 1.107–1.437) and extended median 1.241 (1.134–1.400). The extended-model Emax is shown as a point estimate only for the reason described in Supplementary Methods S2.

Supplementary Table S3. Paired differences between the primary extended and core models

Metric	Mean difference (95% interval)
Average precision	0.037 (-0.010 to 0.069)
Ultrasounds avoided per 1,000 at t90	18.299 (-85.575 to 138.142)
Ultrasounds avoided per 1,000 at t95	32.936 (-85.575 to 180.929)
Brier score	-0.003 (-0.006 to 0.001)
Absolute calibration-slope deviation	-0.004 (-0.178 to 0.107)
Log loss	-0.011 (-0.018 to 0.002)
Net benefit at threshold probability 0.05	0.002 (-0.001 to 0.005)
Net benefit at threshold probability 0.10	0.002 (-0.004 to 0.008)
Net benefit at threshold probability 0.15	0.004 (-0.004 to 0.011)
Net benefit at threshold probability 0.20	0.006 (-0.002 to 0.013)
Precision-recall area under the curve	0.037 (-0.011 to 0.070)
Ultrasound referrals per 1,000 at t90	-18.299 (-138.142 to 85.575)
Ultrasound referrals per 1,000 at t95	-32.936 (-180.929 to 85.575)
ROC area under the curve	0.015 (-0.010 to 0.031)

Differences are primary extended minus core. Intervals containing zero indicate uncertainty in incremental performance.

Supplementary Table S4. Internally validated operating-point accuracy and clinical consequences

Operating point	t90	t95
Target sensitivity	90%	95%
Development threshold	0.084466	0.068444
Sensitivity	89.7% (77.4% to 98.2%)	94.4% (83.7% to 100.0%)
Specificity	38.1% (22.2% to 53.3%)	25.1% (8.5% to 42.1%)
Positive predictive value	21.5% (16.5% to 26.5%)	19.2% (14.9% to 23.8%)
Negative predictive value	95.4% (91.6% to 99.0%)	96.5% (92.2% to 100.0%)
Referral proportion	66.3% (51.7% to 81.0%)	78.0% (61.9% to 92.4%)
Referred per 1,000	663 (517 to 810)	780 (619 to 924)
Avoided per 1,000	337 (190 to 483)	220 (76 to 381)
Detected cases per 1,000	142 (108 to 176)	149 (116 to 183)
Missed cases per 1,000	16 (3 to 37)	9 (0 to 26)
Ultrasounds per detected case	4.719 (3.767 to 6.049)	5.276 (4.210 to 6.707)

Development thresholds were selected from full-cohort repeated cross-fitted predictions. All other values are means with 95% intervals from out-of-bag internal validation of the complete threshold-selection procedure.

Supplementary Table S5. Prespecified sensitivity analyses

Analysis	ROC area under the curve	Precision-recall area under the curve	Brier score	Calibration slope	Mean absolute calibration error
Primary extended ridge	0.769 (0.724 to 0.811)	0.412 (0.329 to 0.496)	0.114 (0.099 to 0.129)	1.086 (0.873 to 1.322)	0.014 (0.007 to 0.039)
Core ridge comparator	0.754 (0.710 to 0.797)	0.376 (0.299 to 0.465)	0.117 (0.102 to 0.132)	1.064 (0.838 to 1.320)	0.020 (0.010 to 0.044)
Extended ridge, lambda.1se	0.771 (0.725 to 0.814)	0.416 (0.333 to 0.500)	0.117 (0.102 to 0.133)	1.841 (1.481 to 2.242)	0.050 (0.032 to 0.071)
Extended ridge, injection frequency as factor	0.770 (0.726 to 0.813)	0.417 (0.336 to 0.500)	0.114 (0.099 to 0.130)	1.089 (0.876 to 1.325)	0.016 (0.007 to 0.040)
Extended unpenalized logistic regression	0.767 (0.723 to 0.809)	0.408 (0.326 to 0.492)	0.115 (0.099 to 0.131)	0.879 (0.706 to 1.070)	0.012 (0.009 to 0.037)
Extended ridge with prespecified natural splines	0.764 (0.719 to 0.806)	0.398 (0.319 to 0.483)	0.115 (0.099 to 0.130)	1.097 (0.881 to 1.338)	0.017 (0.008 to 0.041)
Core ridge with robust scaling	0.753 (0.709 to 0.797)	0.378 (0.301 to 0.462)	0.117 (0.102 to 0.133)	1.018 (0.811 to 1.260)	0.019 (0.009 to 0.043)
Extended ridge with robust scaling	0.768 (0.725 to 0.812)	0.408 (0.326 to 0.493)	0.114 (0.099 to 0.130)	1.036 (0.841 to 1.267)	0.014 (0.007 to 0.038)

Values are repeated nested cross-fitted estimates with patient-bootstrap 95% percentile intervals.

Supplementary figures

Supplementary Figure S1. Cohort characteristics and predictor distributions. (A) Standardized differences. (B) Observed distributions. (C) Ultrasound-positive proportions with Wilson intervals. (D) Descriptive associations between variables of different measurement types. BMI, body mass index; CRP, C-reactive protein; eGFR, estimated glomerular filtration rate; HbA1c, glycated hemoglobin; qd, once daily; bid, twice daily; tid, three times daily.

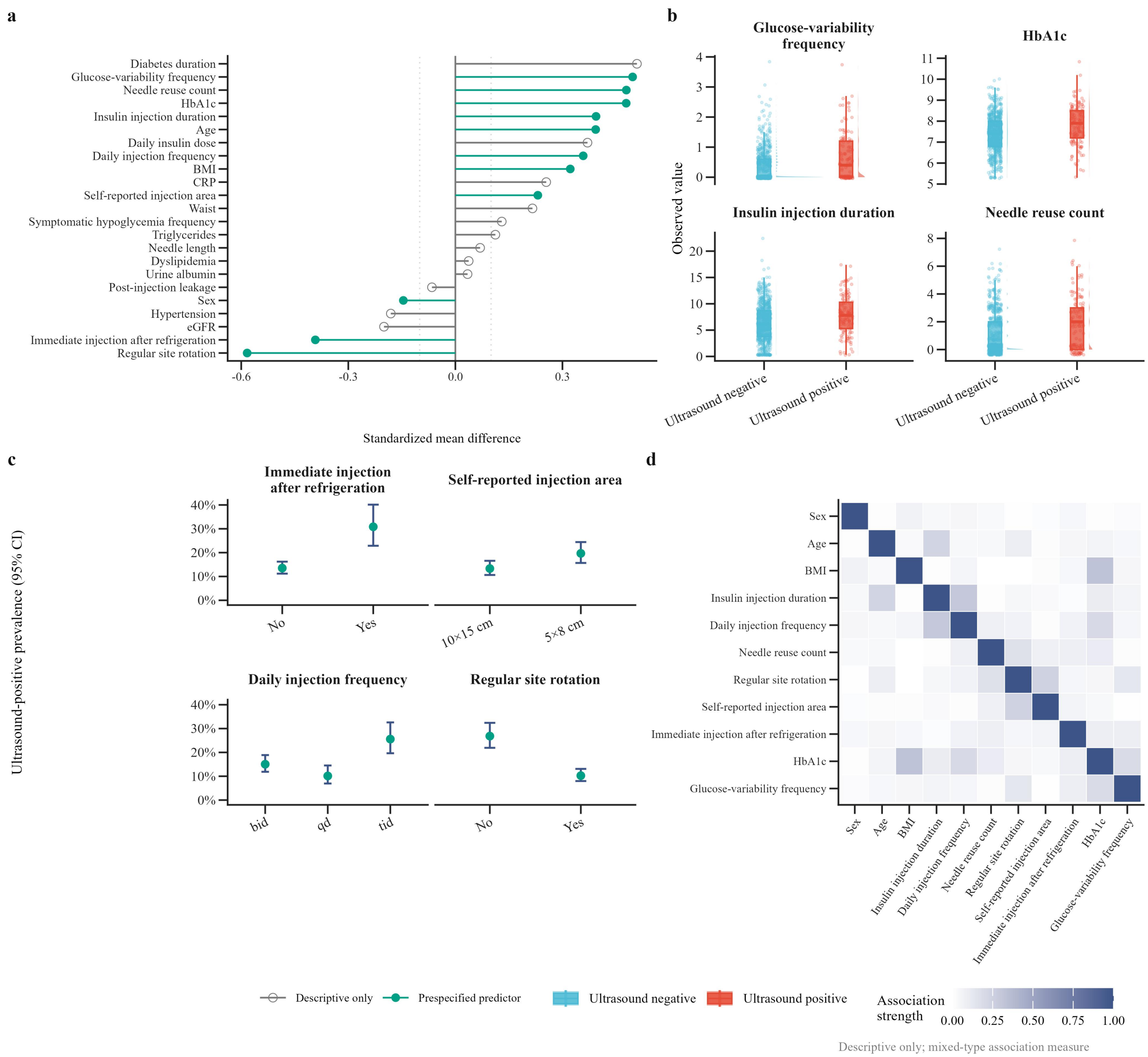

Supplementary Figure S2. Model coefficients and incremental information. (A) Standardized ridge coefficients. (B) Bootstrap coefficient stability intervals. (C) Changes in patient-level probabilities. (D) Paired differences in model performance. Coefficients and stability intervals summarize the fitted penalized models. Irregular site rotation is coded 1 versus regular rotation coded 0; the descriptive label in Table 1 and Supplementary Figure S1 refers to regular site rotation.

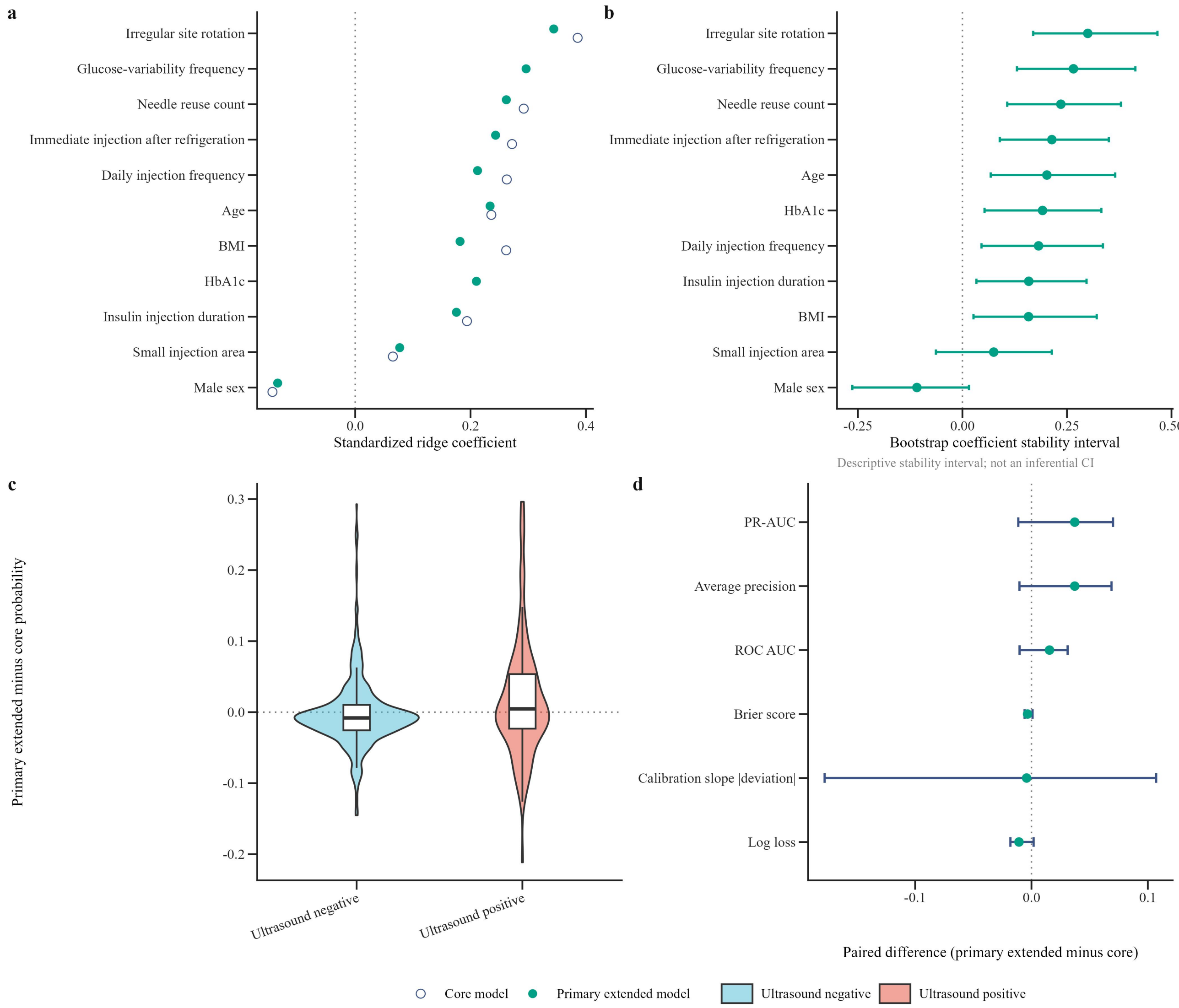

Supplementary Figure S3. Clinical utility of selective ultrasound. (A) Decision curves. (B) Net reduction in ultrasound examinations per 100 patients. (C) Referrals and true-positive detections per 1,000 patients. (D) Ultrasound examinations avoided and cases missed across threshold probabilities. The curves use cross-fitted probabilities; the positive decision is referral for high-frequency ultrasound. Net benefit is evaluated over threshold probabilities of 0.02–0.30.

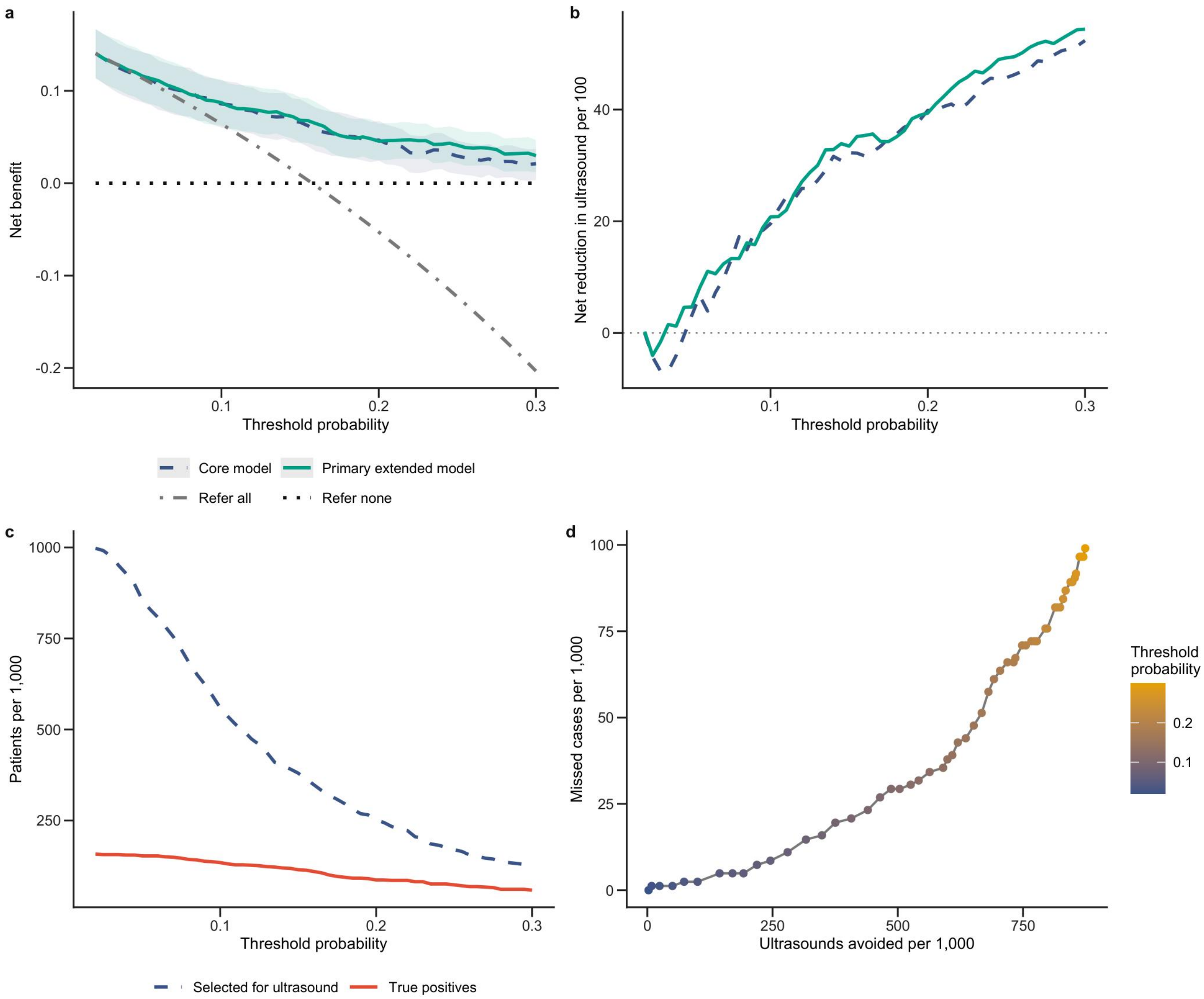

Supplementary Figure S4. High-sensitivity operating points. (A) Sensitivity and specificity across probability thresholds. (B) Distributions of bootstrap-developed thresholds. (C) Clinical consequences per 1,000 patients at t90 and t95. (D) Out-of-bag estimates and 95% intervals for sensitivity, specificity, positive predictive value, and negative predictive value. Development thresholds use cross-fitted predictions; accuracy and clinical-consequence estimates use out-of-bag validation of the complete threshold-selection procedure. FN, false negatives; FP, false positives; TN, true negatives; TP, true positives.

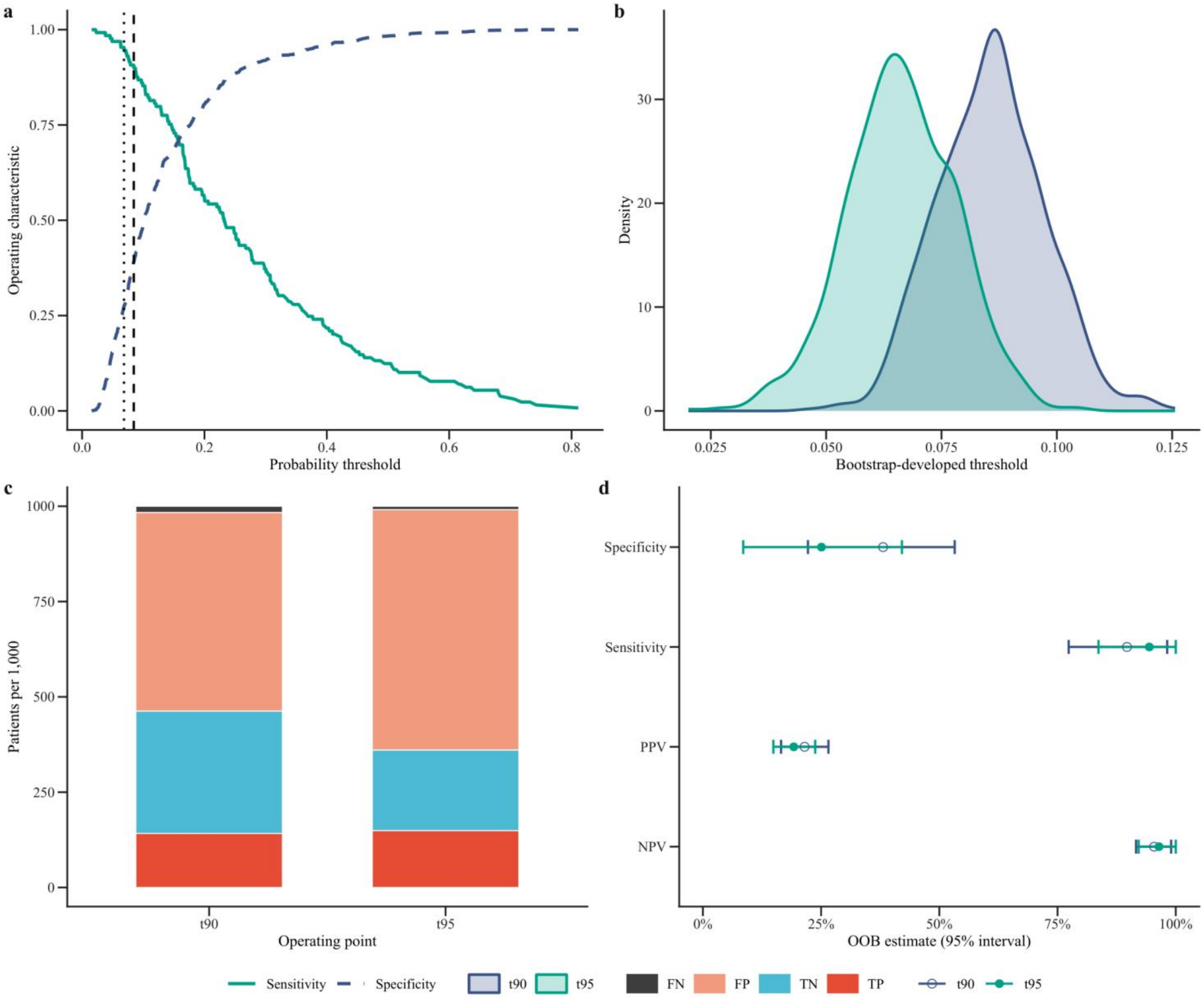

Other reporting information

The local modeling analysis plan was specified before formal model fitting but was not publicly deposited. Patients and members of the public were not involved in study design, conduct, analysis, reporting, or dissemination. Individual-level data and analysis/model code are not publicly shared.
