# Supplementary material for: Diagnostic triage for ultrasound-detected subclinical insulin injection–related lipohypertrophy in older adults with diabetes: development and internal validation
Source: Front Endocrinol (Lausanne). 2026 Oct 21;17:1997042. doi: 10.3389/fendo.2026.1997042 (PMC13730916; doi:10.3389/fendo.2026.1997042)
Supplement: Supplementary file 2 [file DataSheet2.pdf]

Supplementary Figure S1, panel A

a

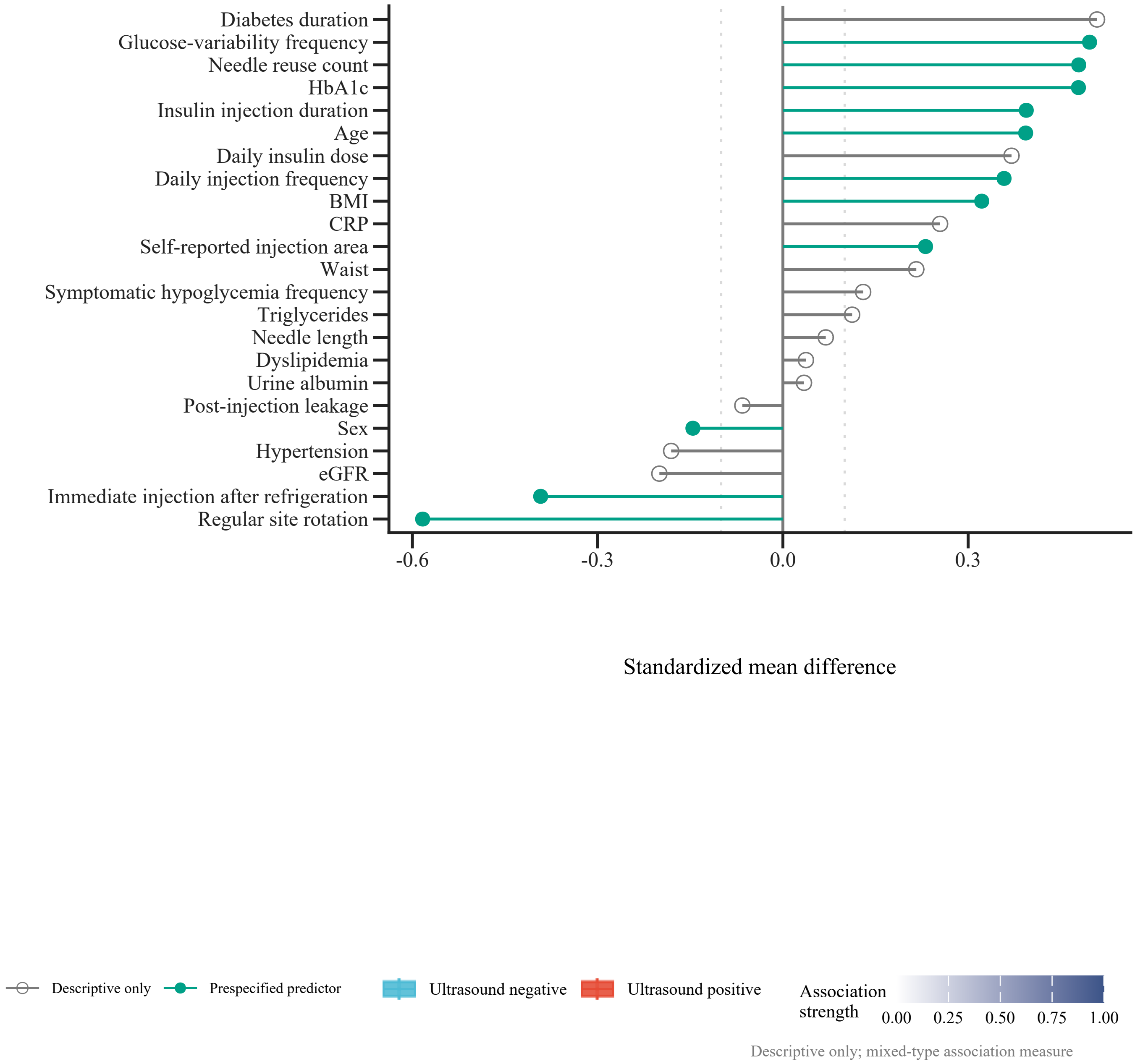

Supplementary Figure S1. Cohort characteristics and predictor distributions. (A) Standardized differences. (B) Observed distributions. (C) Ultrasound-positive proportions with Wilson intervals. (D) Descriptive associations between variables of different measurement types. BMI, body mass index; CRP, C-reactive protein; eGFR, estimated glomerular filtration rate; HbA1c, glycated hemoglobin; qd, once daily; bid, twice daily; tid, three times daily.

Supplementary Figure S1, panel B

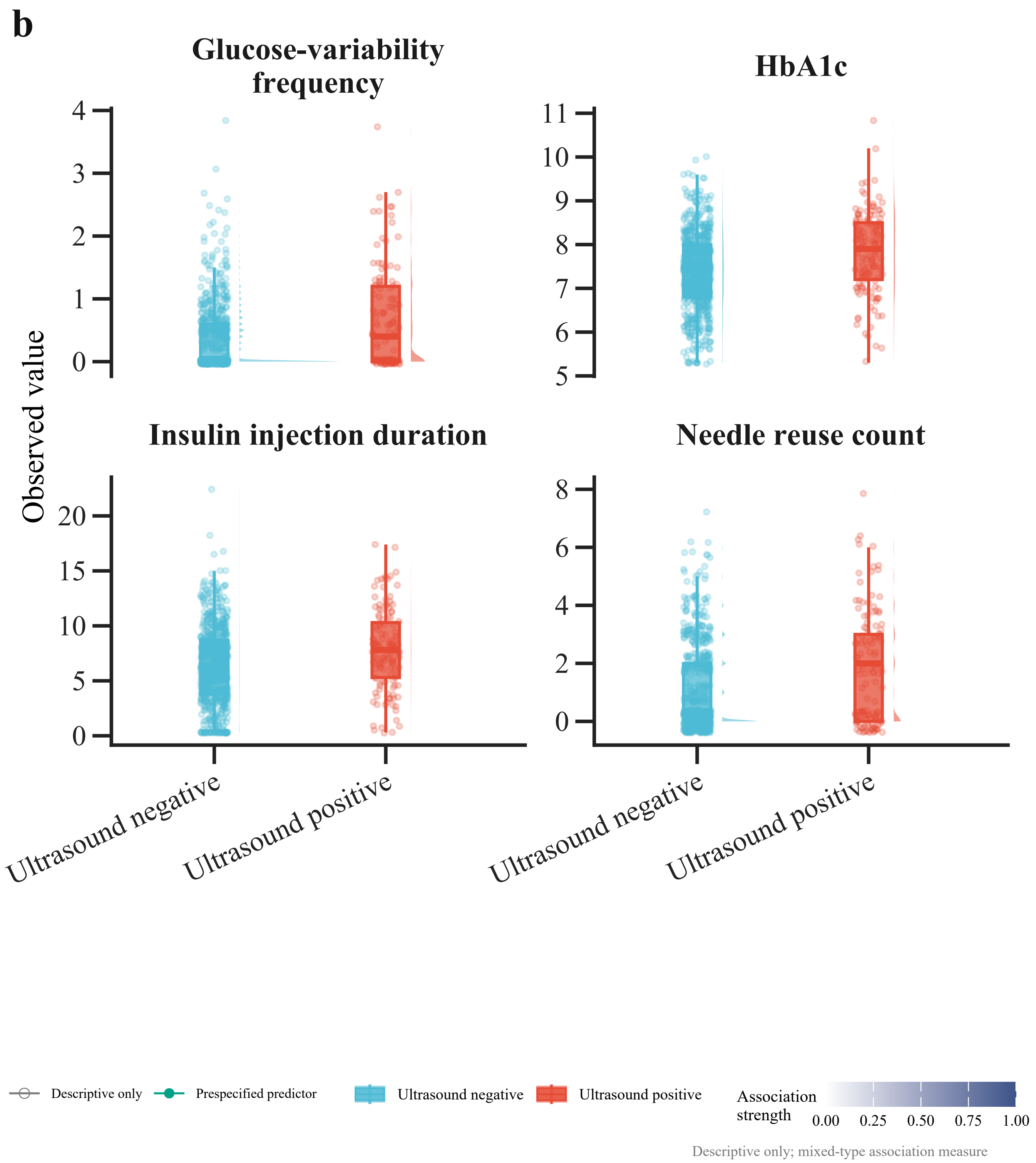

Supplementary Figure S1. Cohort characteristics and predictor distributions. (A) Standardized differences. (B) Observed distributions. (C) Ultrasound-positive proportions with Wilson intervals. (D) Descriptive associations between variables of different measurement types. BMI, body mass index; CRP, C-reactive protein; eGFR, estimated glomerular filtration rate; HbA1c, glycated hemoglobin; qd, once daily; bid, twice daily; tid, three times daily.

Supplementary Figure S1, panel C

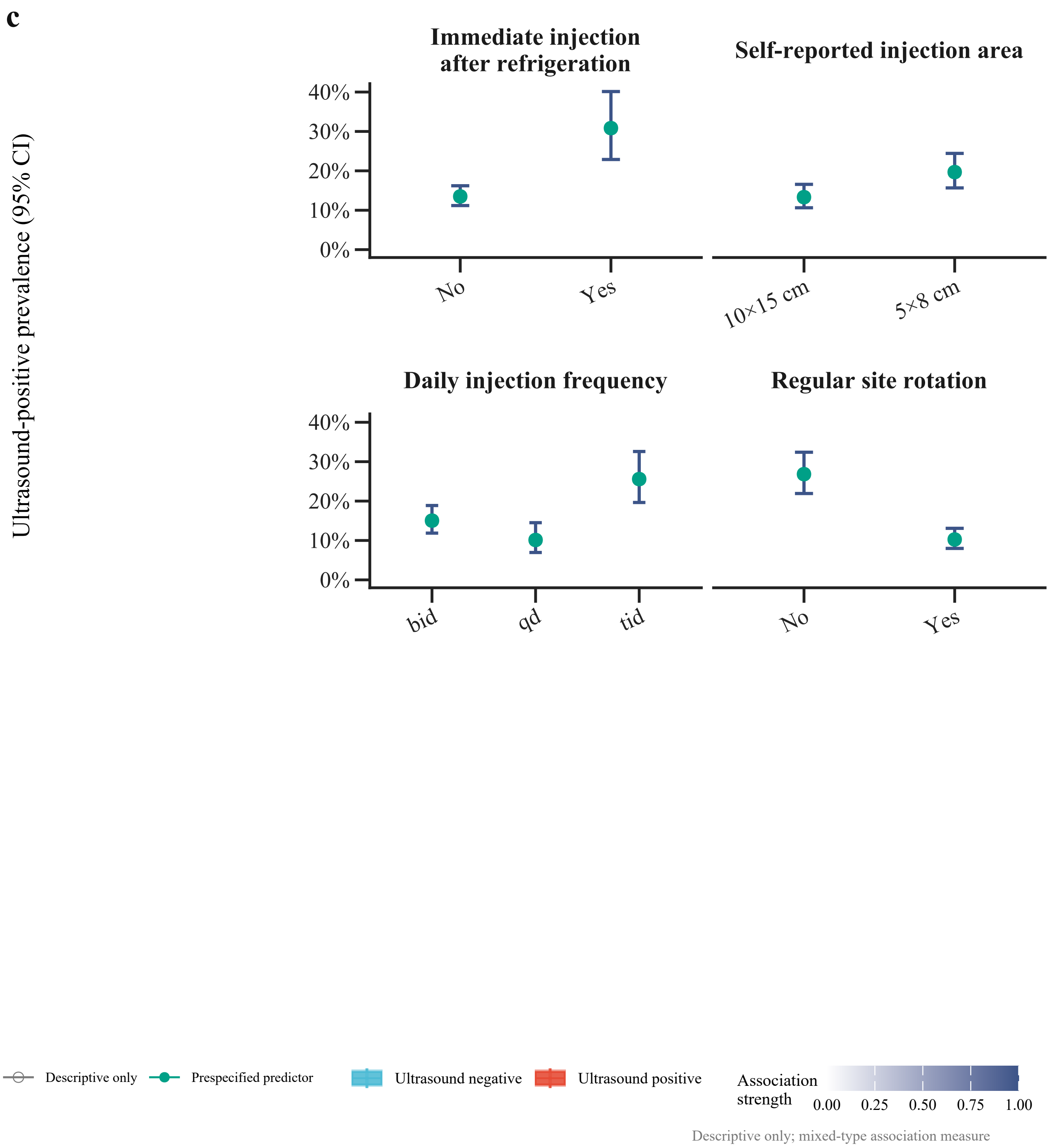

Supplementary Figure S1. Cohort characteristics and predictor distributions. (A) Standardized differences. (B) Observed distributions. (C) Ultrasound-positive proportions with Wilson intervals. (D) Descriptive associations between variables of different measurement types. BMI, body mass index; CRP, C-reactive protein; eGFR, estimated glomerular filtration rate; HbA1c, glycated hemoglobin; qd, once daily; bid, twice daily; tid, three times daily.

Supplementary Figure S1, panel D

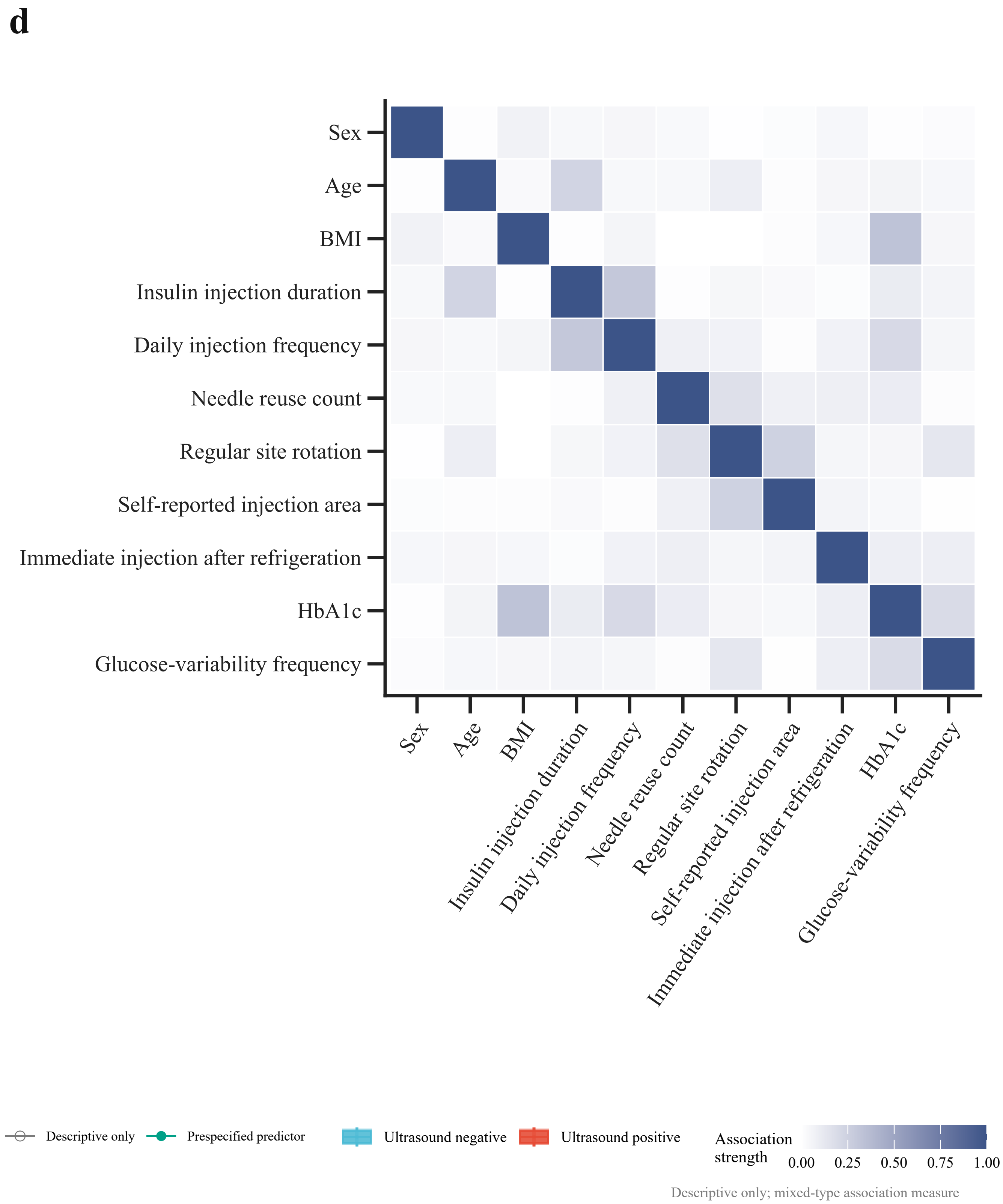

Supplementary Figure S1. Cohort characteristics and predictor distributions. (A) Standardized differences. (B) Observed distributions. (C) Ultrasound-positive proportions with Wilson intervals. (D) Descriptive associations between variables of different measurement types. BMI, body mass index; CRP, C-reactive protein; eGFR, estimated glomerular filtration rate; HbA1c, glycated hemoglobin; qd, once daily; bid, twice daily; tid, three times daily.

Supplementary Figure S2, panel A

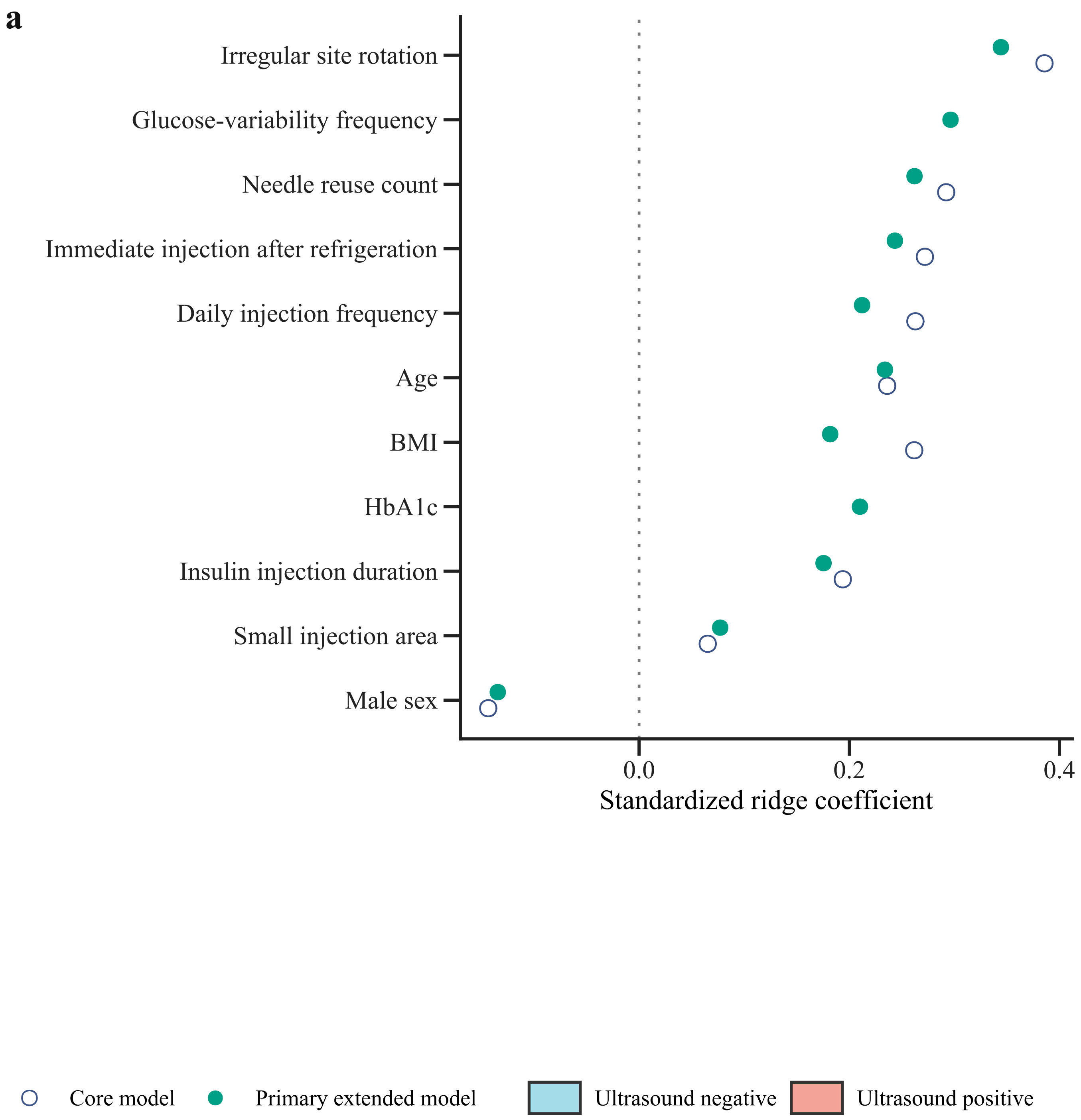

Supplementary Figure S2. Model coefficients and incremental information. (A) Standardized ridge coefficients. (B) Bootstrap coefficient stability intervals. (C) Changes in patient-level probabilities. (D) Paired differences in model performance. Coefficients and stability intervals summarize the fitted penalized models. Irregular site rotation is coded 1 versus regular rotation coded 0; the descriptive label in Table 1 and Supplementary Figure S1 refers to regular site rotation.

Supplementary Figure S2, panel B

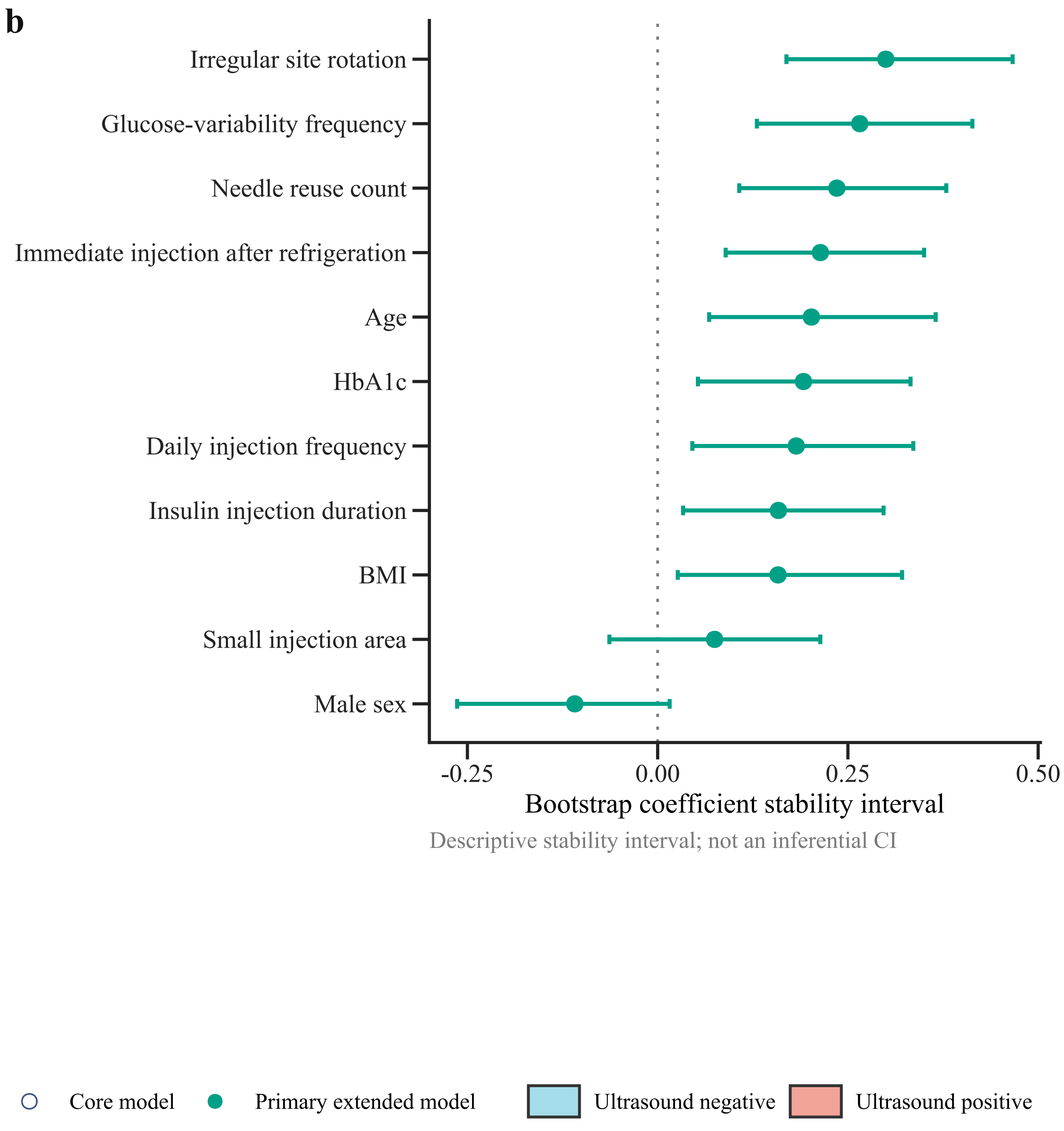

Supplementary Figure S2. Model coefficients and incremental information. (A) Standardized ridge coefficients. (B) Bootstrap coefficient stability intervals. (C) Changes in patient-level probabilities. (D) Paired differences in model performance. Coefficients and stability intervals summarize the fitted penalized models. Irregular site rotation is coded 1 versus regular rotation coded 0; the descriptive label in Table 1 and Supplementary Figure S1 refers to regular site rotation.

Supplementary Figure S2, panel C

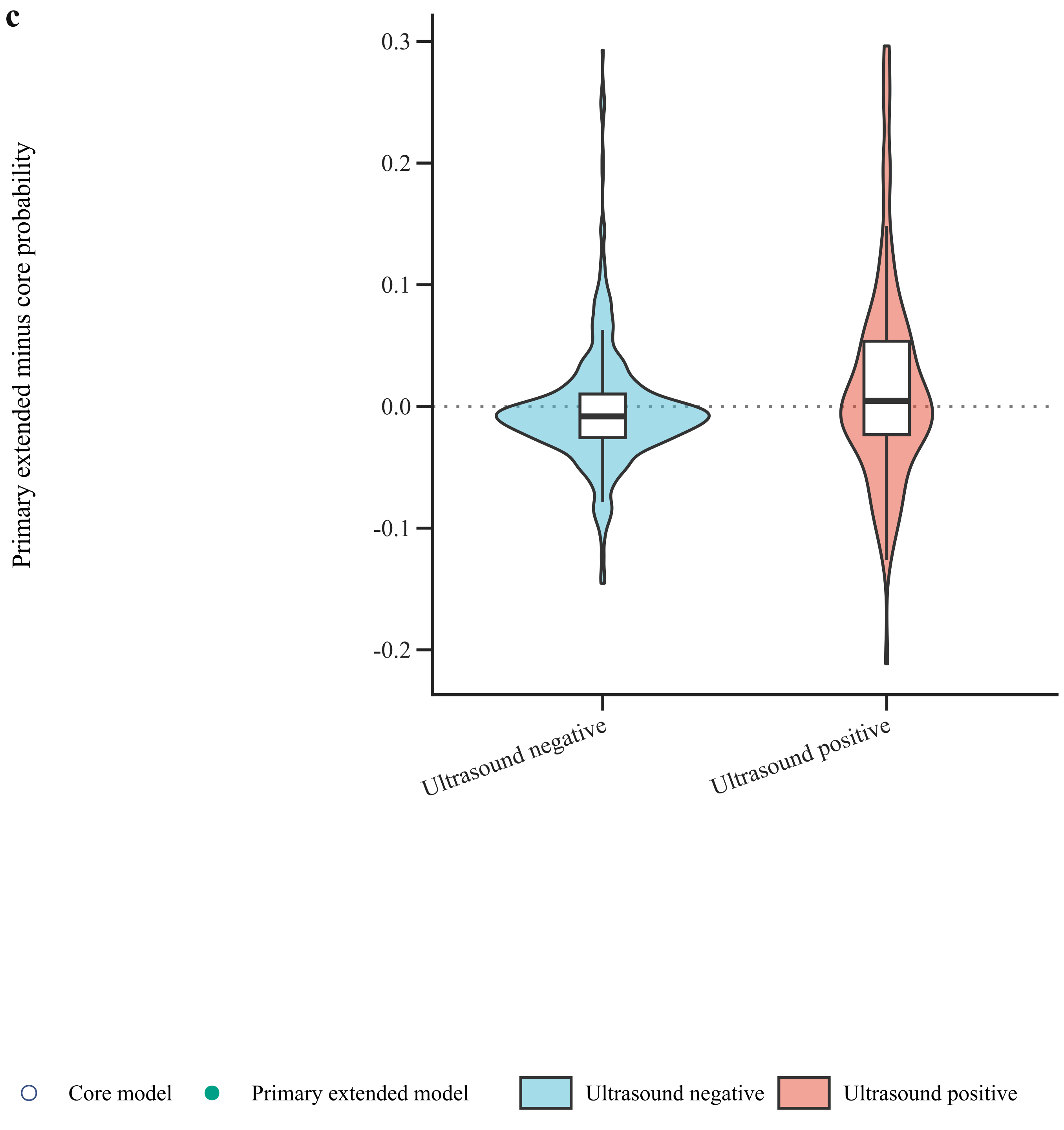

Supplementary Figure S2. Model coefficients and incremental information. (A) Standardized ridge coefficients. (B) Bootstrap coefficient stability intervals. (C) Changes in patient-level probabilities. (D) Paired differences in model performance. Coefficients and stability intervals summarize the fitted penalized models. Irregular site rotation is coded 1 versus regular rotation coded 0; the descriptive label in Table 1 and Supplementary Figure S1 refers to regular site rotation.

Supplementary Figure S2, panel D

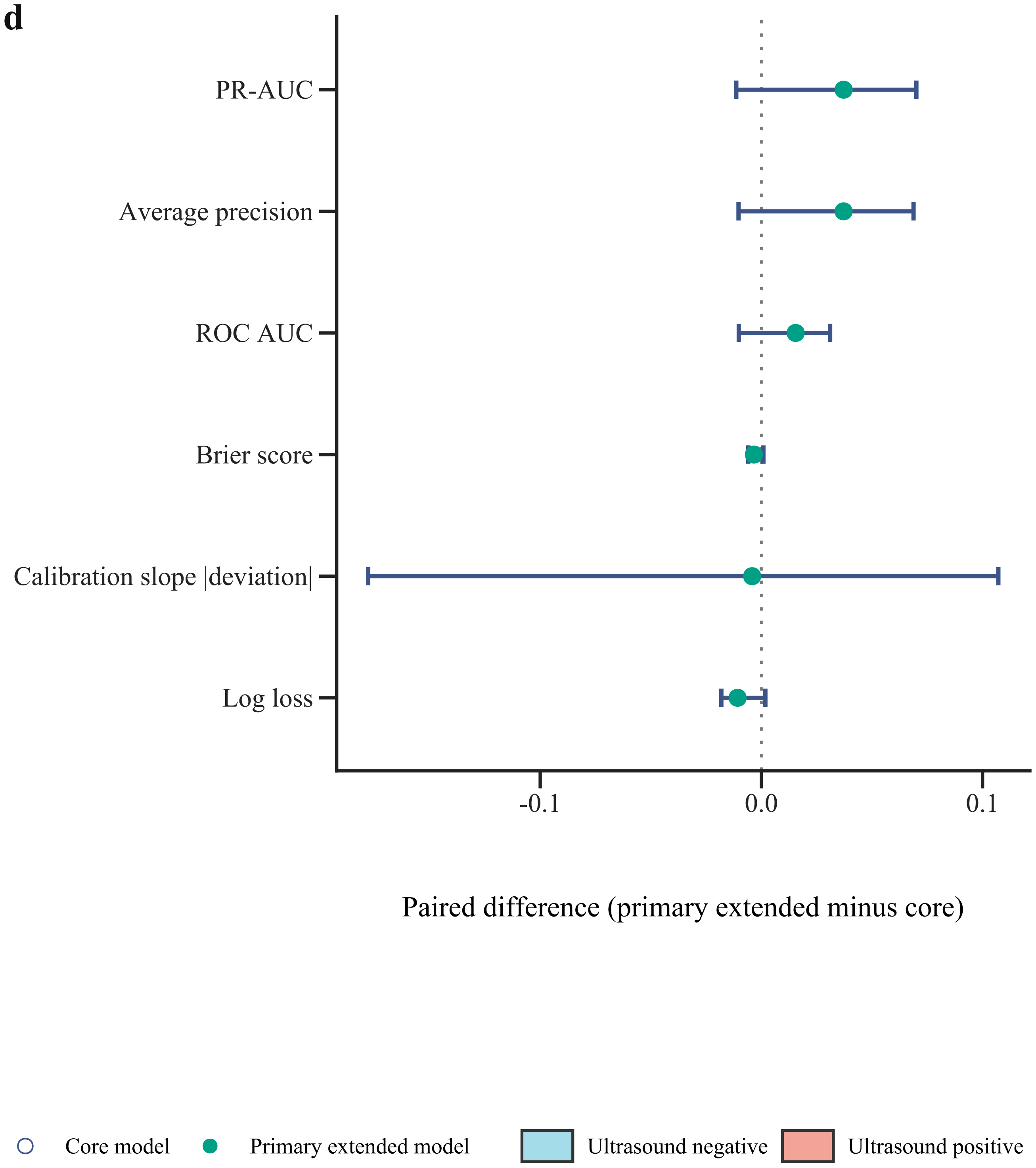

Supplementary Figure S2. Model coefficients and incremental information. (A) Standardized ridge coefficients. (B) Bootstrap coefficient stability intervals. (C) Changes in patient-level probabilities. (D) Paired differences in model performance. Coefficients and stability intervals summarize the fitted penalized models. Irregular site rotation is coded 1 versus regular rotation coded 0; the descriptive label in Table 1 and Supplementary Figure S1 refers to regular site rotation.

Supplementary Figure S3, panel A

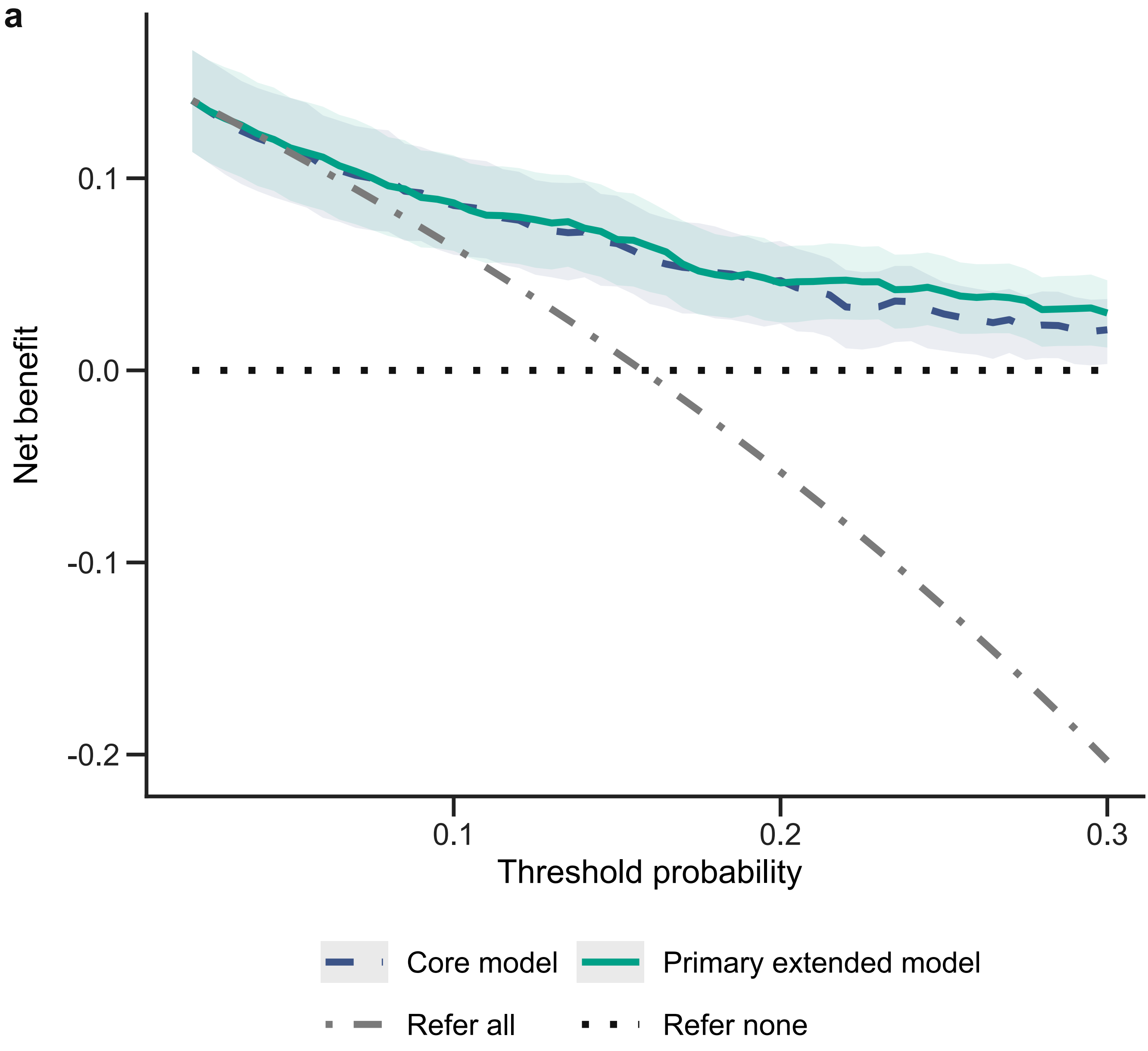

Supplementary Figure S3. Clinical utility of selective ultrasound. (A) Decision curves. (B) Net reduction in ultrasound examinations per 100 patients. (C) Referrals and true-positive detections per 1,000 patients. (D) Ultrasound examinations avoided and cases missed across threshold probabilities. The curves use cross-fitted probabilities; the positive decision is referral for high-frequency ultrasound. Net benefit is evaluated over threshold probabilities of 0.02-0.30.

Supplementary Figure S3, panel B

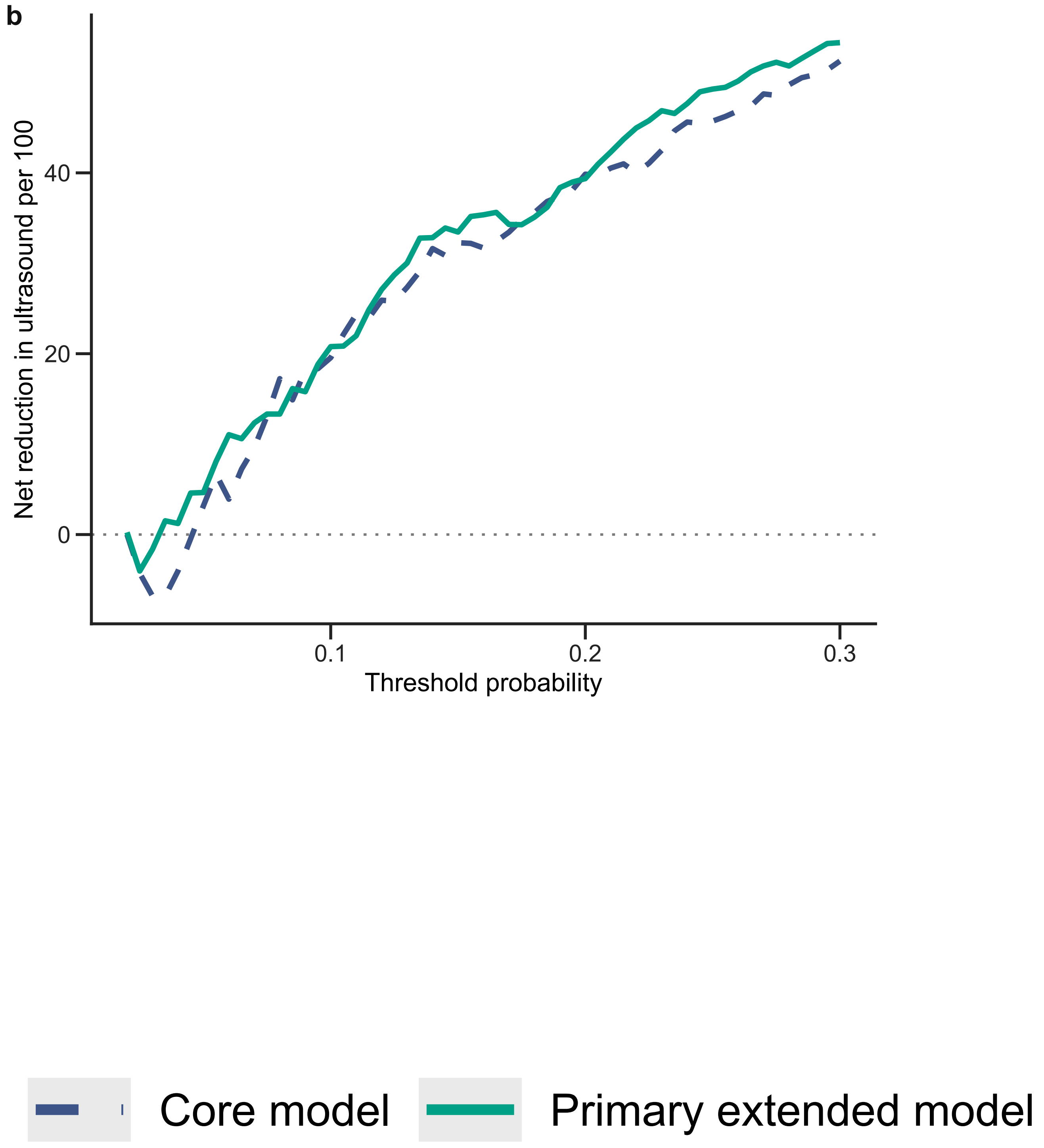

Supplementary Figure S3. Clinical utility of selective ultrasound. (A) Decision curves. (B) Net reduction in ultrasound examinations per 100 patients. (C) Referrals and true-positive detections per 1,000 patients. (D) Ultrasound examinations avoided and cases missed across threshold probabilities. The curves use cross-fitted probabilities; the positive decision is referral for high-frequency ultrasound. Net benefit is evaluated over threshold probabilities of 0.02-0.30.

Supplementary Figure S3, panel C

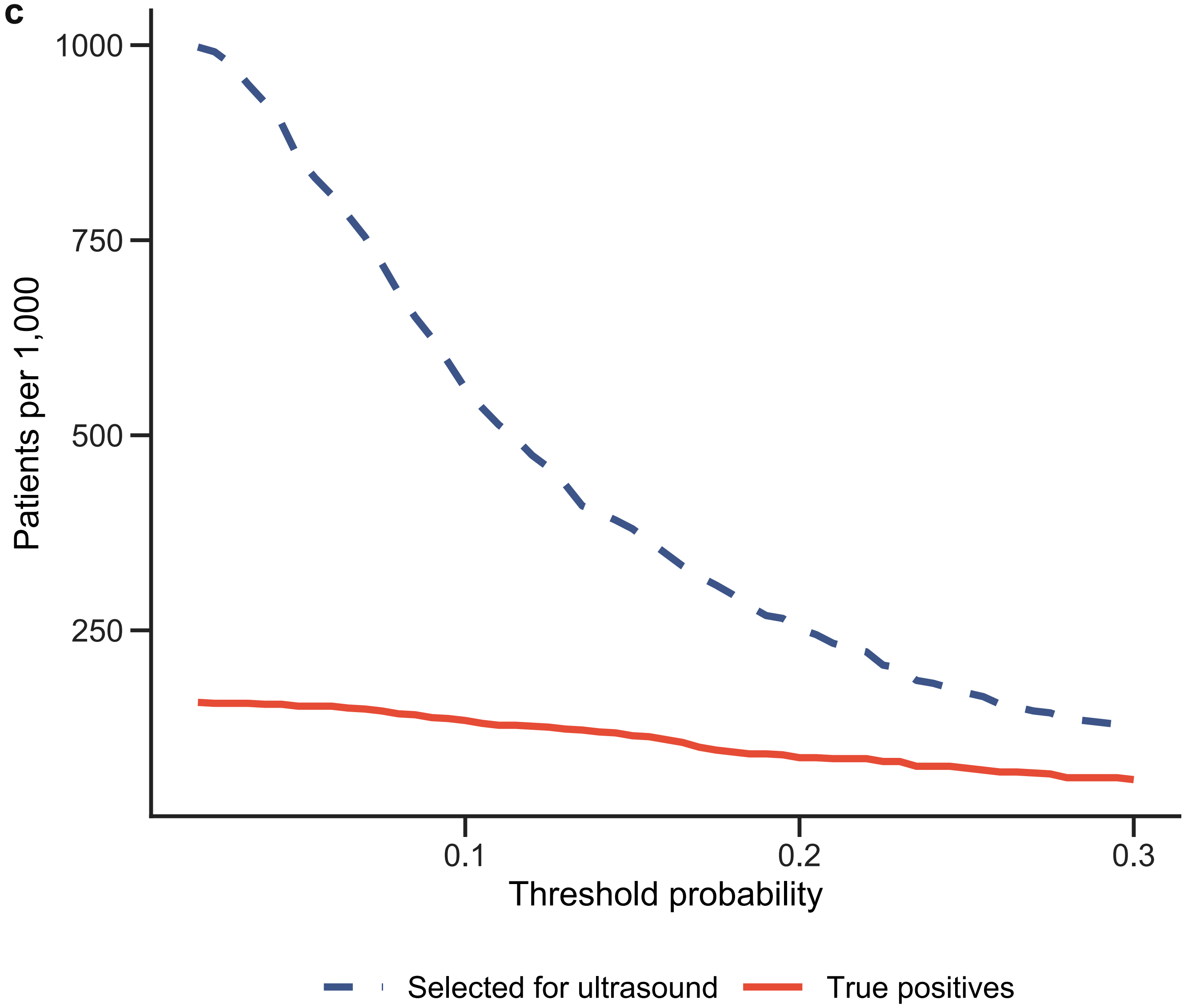

Supplementary Figure S3. Clinical utility of selective ultrasound. (A) Decision curves. (B) Net reduction in ultrasound examinations per 100 patients. (C) Referrals and true-positive detections per 1,000 patients. (D) Ultrasound examinations avoided and cases missed across threshold probabilities. The curves use cross-fitted probabilities; the positive decision is referral for high-frequency ultrasound. Net benefit is evaluated over threshold probabilities of 0.02-0.30.

Supplementary Figure S3, panel D

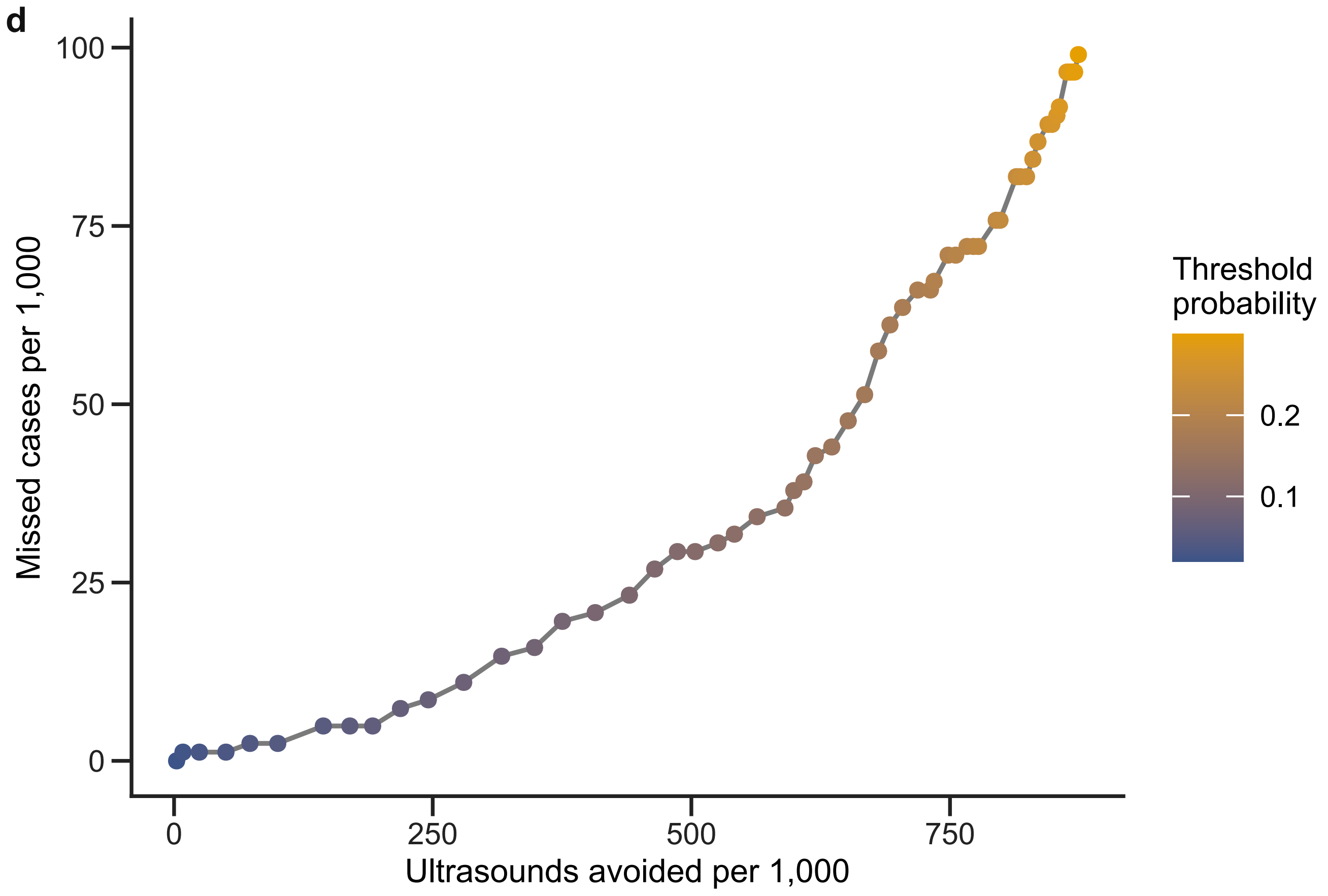

Supplementary Figure S3. Clinical utility of selective ultrasound. (A) Decision curves. (B) Net reduction in ultrasound examinations per 100 patients. (C) Referrals and true-positive detections per 1,000 patients. (D) Ultrasound examinations avoided and cases missed across threshold probabilities. The curves use cross-fitted probabilities; the positive decision is referral for high-frequency ultrasound. Net benefit is evaluated over threshold probabilities of 0.02-0.30.

Supplementary Figure S4, panel A

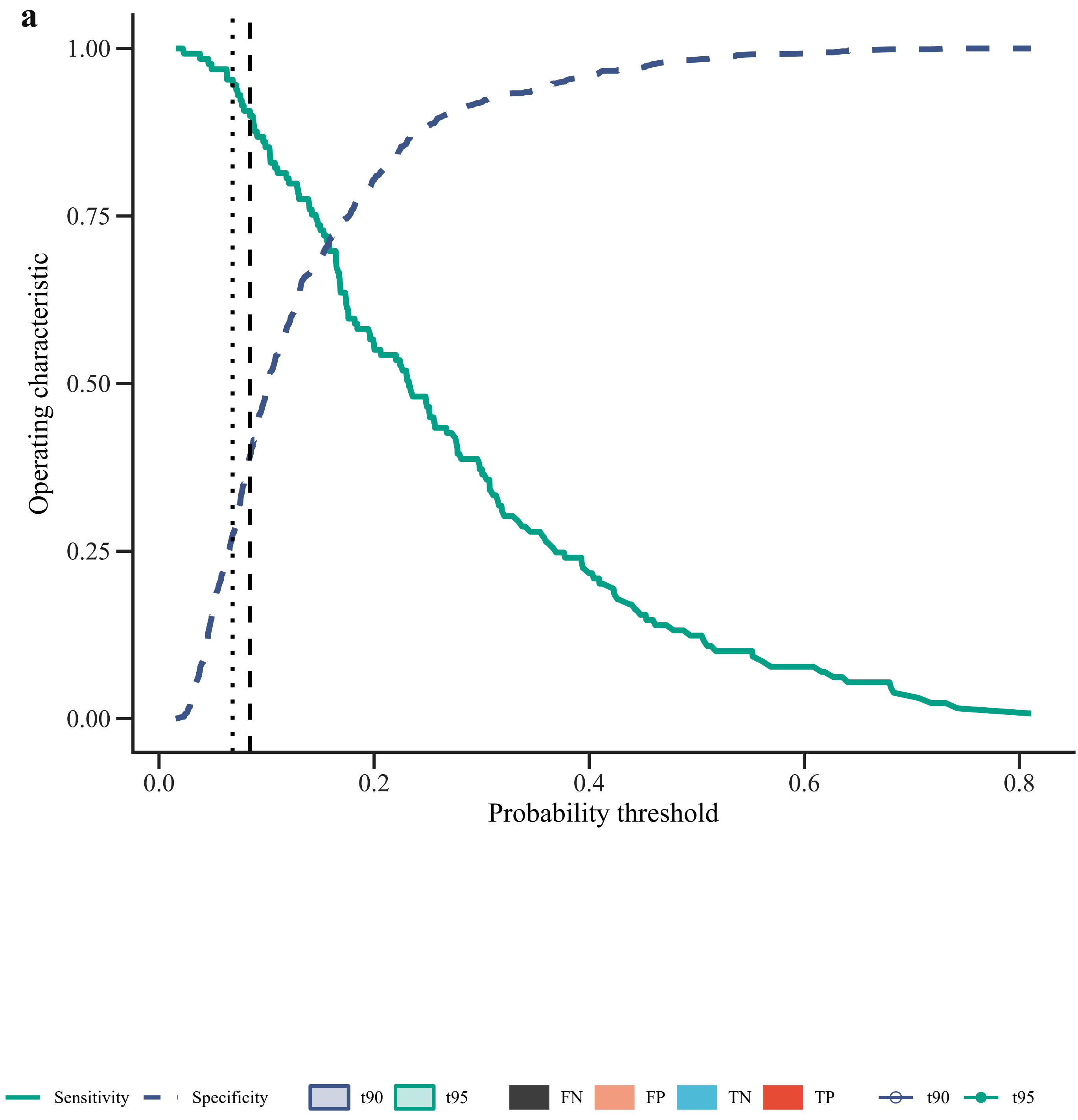

Supplementary Figure S4. High-sensitivity operating points. (A) Sensitivity and specificity across probability thresholds. (B) Distributions of bootstrap-developed thresholds. (C) Clinical consequences per 1,000 patients at t90 and t95. (D) Out-of-bag estimates and 95% intervals for sensitivity, specificity, positive predictive value, and negative predictive value. Development thresholds use cross-fitted predictions; accuracy and clinical-consequence estimates use out-of-bag validation of the complete threshold-selection procedure. FN, false negatives; FP, false positives; TN, true negatives; TP, true positives.

Supplementary Figure S4, panel B

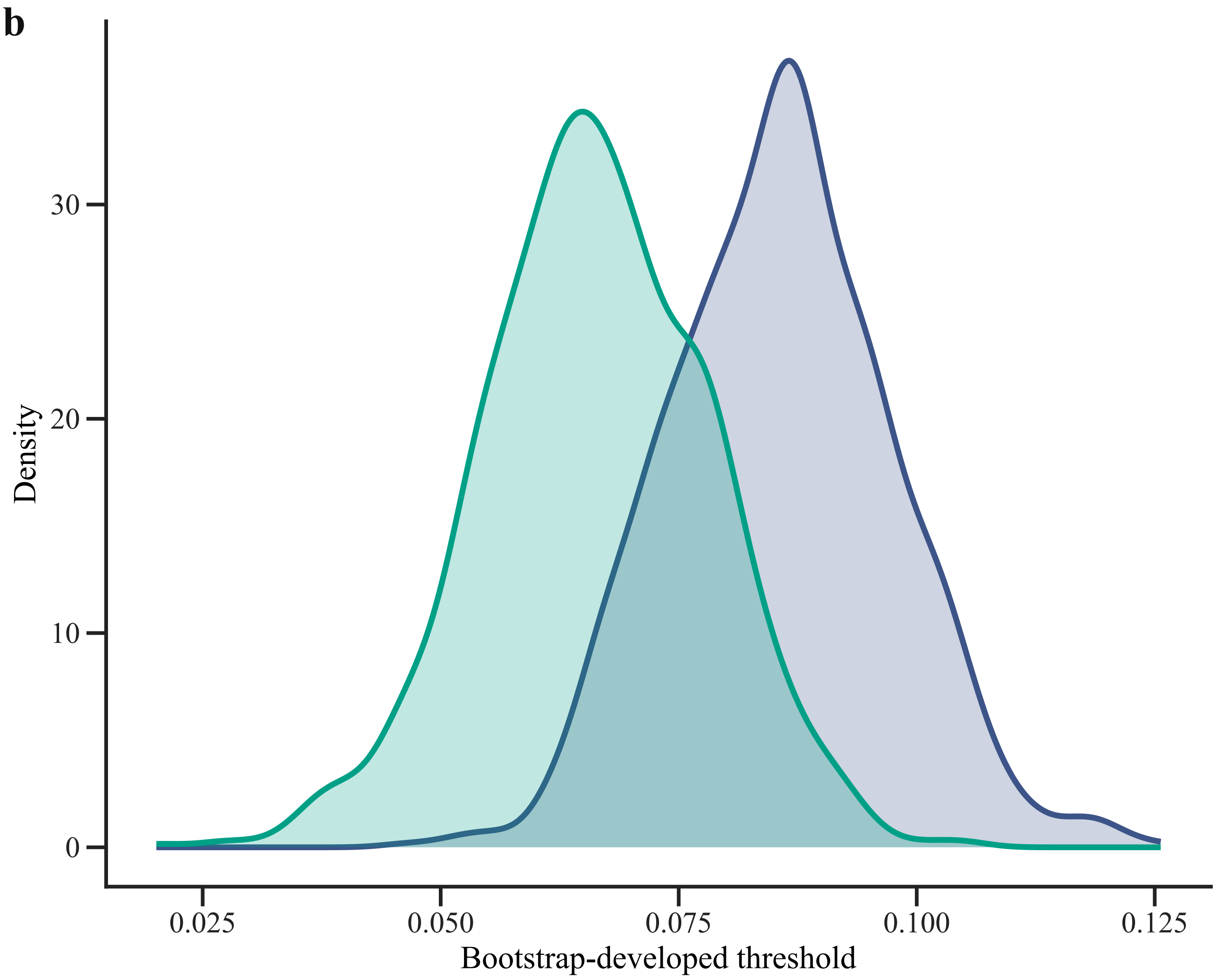

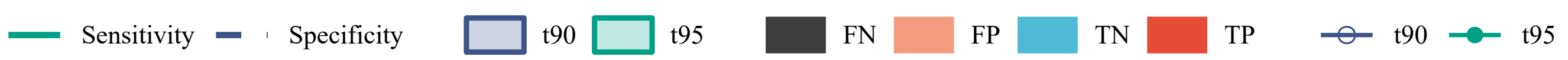

Supplementary Figure S4. High-sensitivity operating points. (A) Sensitivity and specificity across probability thresholds. (B) Distributions of bootstrap-developed thresholds. (C) Clinical consequences per 1,000 patients at t90 and t95. (D) Out-of-bag estimates and 95% intervals for sensitivity, specificity, positive predictive value, and negative predictive value. Development thresholds use cross-fitted predictions; accuracy and clinical-consequence estimates use out-of-bag validation of the complete threshold-selection procedure. FN, false negatives; FP, false positives; TN, true negatives; TP, true positives.

Supplementary Figure S4, panel C

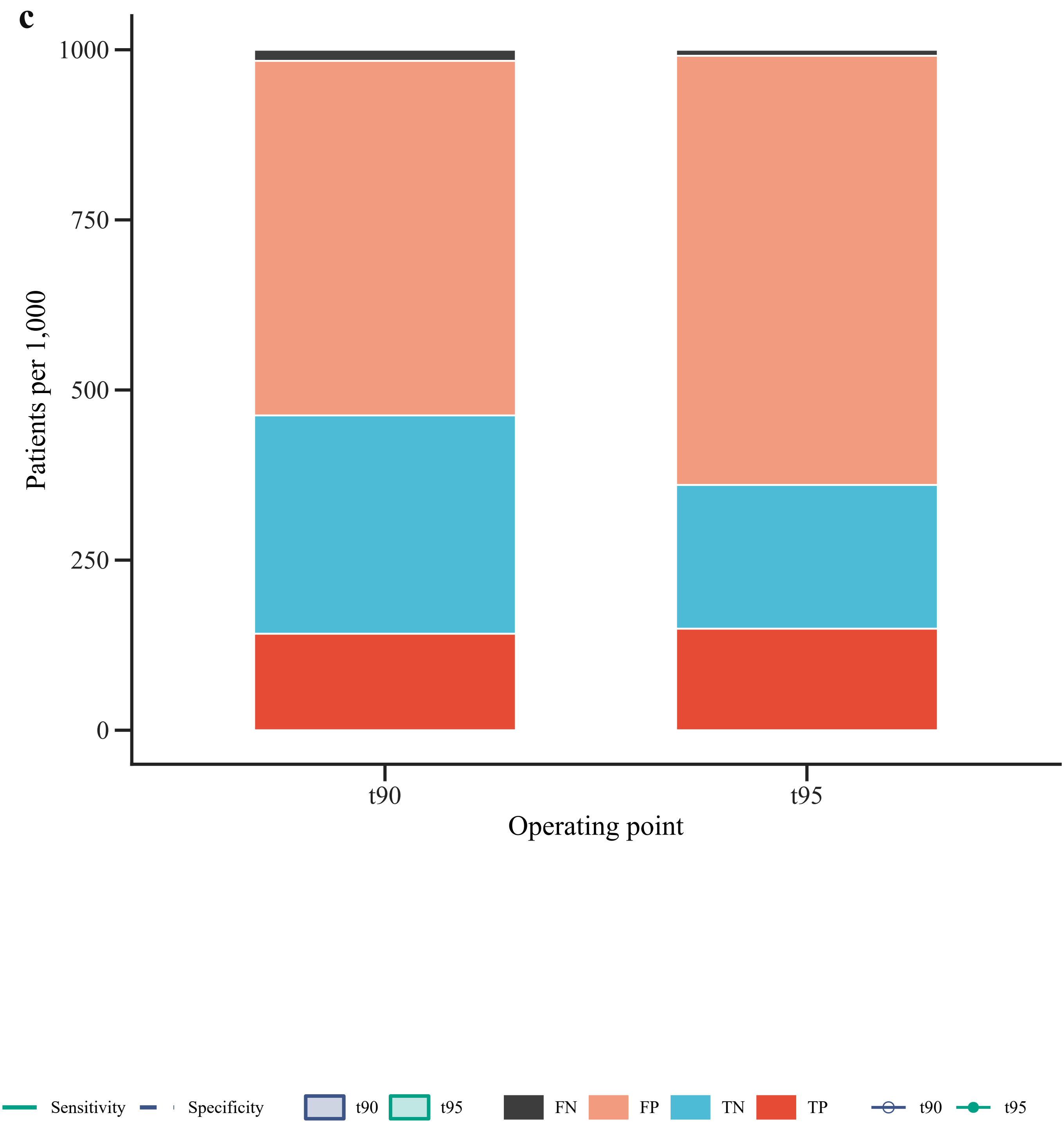

Supplementary Figure S4. High-sensitivity operating points. (A) Sensitivity and specificity across probability thresholds. (B) Distributions of bootstrap-developed thresholds. (C) Clinical consequences per 1,000 patients at t90 and t95. (D) Out-of-bag estimates and 95% intervals for sensitivity, specificity, positive predictive value, and negative predictive value. Development thresholds use cross-fitted predictions; accuracy and clinical-consequence estimates use out-of-bag validation of the complete threshold-selection procedure. FN, false negatives; FP, false positives; TN, true negatives; TP, true positives.

Supplementary Figure S4, panel D

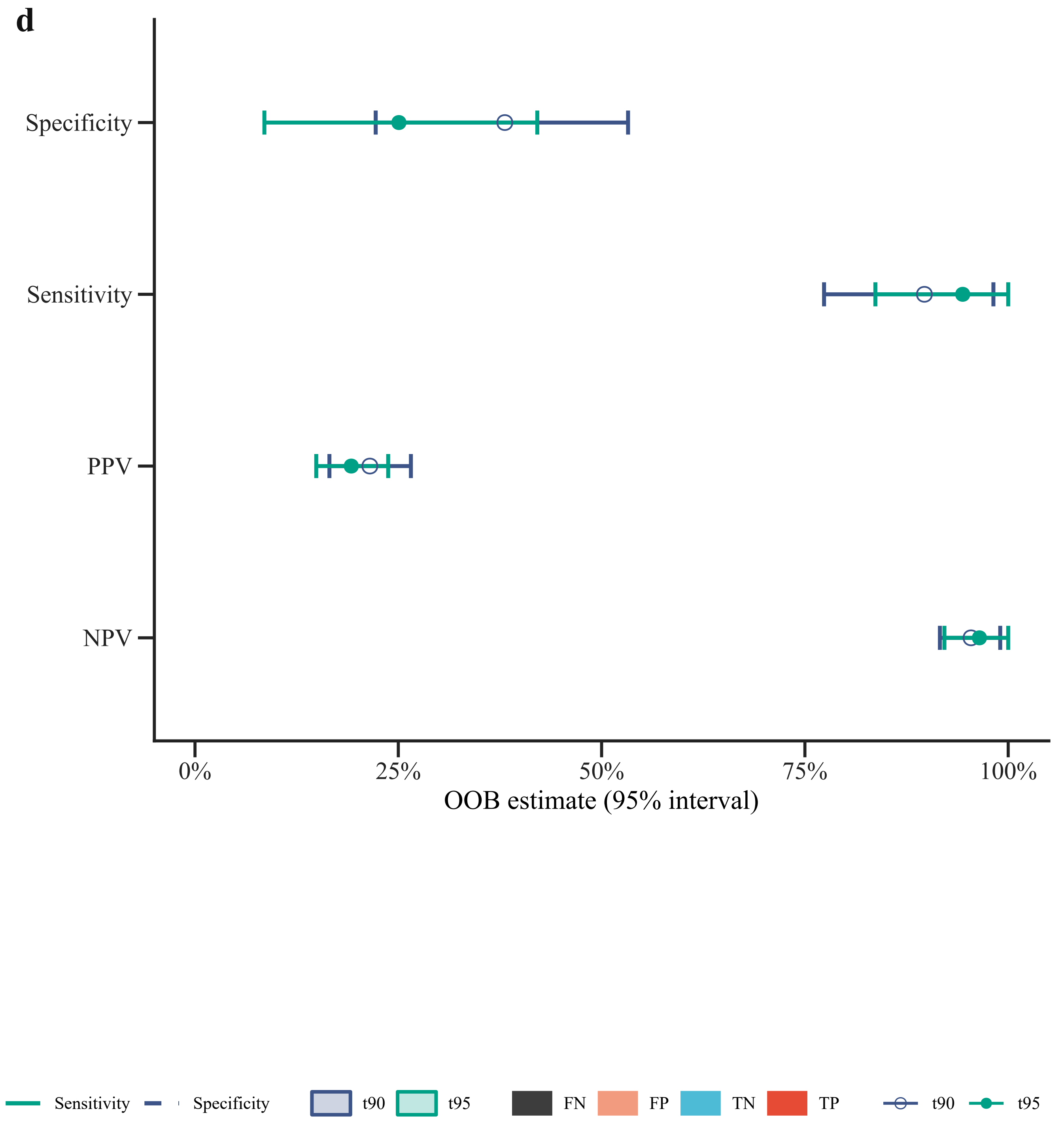

Supplementary Figure S4. High-sensitivity operating points. (A) Sensitivity and specificity across probability thresholds. (B) Distributions of bootstrap-developed thresholds. (C) Clinical consequences per 1,000 patients at t90 and t95. (D) Out-of-bag estimates and 95% intervals for sensitivity, specificity, positive predictive value, and negative predictive value. Development thresholds use cross-fitted predictions; accuracy and clinical-consequence estimates use out-of-bag validation of the complete threshold-selection procedure. FN, false negatives; FP, false positives; TN, true negatives; TP, true positives.
